# Brain MR image segmentation based on an improved active contour model

**DOI:** 10.1371/journal.pone.0183943

**Published:** 2017-08-30

**Authors:** Xiangrui Meng, Wenya Gu, Yunjie Chen, Jianwei Zhang

**Affiliations:** 1 School of Binjiang, Nanjing University of Information Science and Technology, Nanjing, CHINA; 2 School of math and statistics, Nanjing University of Information Science and Technology, Nanjing, CHINA; Beijing University of Technology, CHINA

## Abstract

It is often a difficult task to accurately segment brain magnetic resonance (MR) images with intensity in-homogeneity and noise. This paper introduces a novel level set method for simultaneous brain MR image segmentation and intensity inhomogeneity correction. To reduce the effect of noise, novel anisotropic spatial information, which can preserve more details of edges and corners, is proposed by incorporating the inner relationships among the neighbor pixels. Then the proposed energy function uses the multivariate Student's t-distribution to fit the distribution of the intensities of each tissue. Furthermore, the proposed model utilizes Hidden Markov random fields to model the spatial correlation between neigh-boring pixels/voxels. The means of the multivariate Student's t-distribution can be adaptively estimated by multiplying a bias field to reduce the effect of intensity inhomogeneity. In the end, we reconstructed the energy function to be convex and calculated it by using the Split Bregman method, which allows our framework for random initialization, thereby allowing fully automated applications. Our method can obtain the final result in less than 1 second for 2D image with size 256 × 256 and less than 300 seconds for 3D image with size 256 × 256 × 171. The proposed method was compared to other state-of-the-art segmentation methods using both synthetic and clinical brain MR images and increased the accuracies of the results more than 3%.

## Introduction

Brain disease has become one of the most talked-about diseases in the world. Scientists mainly depend on medical imaging technologies to analyze brain diseases. The use of magnetic resonance imaging has become a preferred choice of method because it is generally painless, harmless and can provide very informative diagnostic images of most of the relevant organs and tissues. Among the MRI (Magnetic resonance image) data analysis, precise measurement of the distribution of tissues of interest (TOI), including gray matter (GM), white matter (WM) and cerebrospinal fluid (CSF), plays an important role for brain studies. In order to obtain the precise measurement, accurate image segmentation is a crucial step. Although many segmentation methods have been reported [1], automated and accurate segmentation still remains a difficult task due to the noise and intensity inhomogeneity (also named as bias field), which can easily be found in MRI.

Among the proposed segmentation methods, active contour models (also named as snake models) [2, 3] in particular have been widely used. The active contour models can provide smooth and closed contours as segmentation results, can be incorporated by using various prior knowledge, such as shape prior information and intensity distribution, and can achieve sub-pixel accuracy of the boundaries of TOI [4,5]. However, the snake model is local optima and topology invariable. Furthermore, different parameters should be set when segmenting different images [6]. In order to deal with these problems, level set methods [7, 8] have been proposed and widely further improved.

The existing level set methods can be categorized into two categories: edge-based models [9–12] and region-based models [4, 5, 13–17]. Edge-based models utilize local edge information, such as gradients of edge pixels, to attract the evolving contour/surface toward the boundaries of the region of interest (ROI). These methods are based on local edge information, which makes them sensitive to the noise, low contrast and hard to obtain satisfied results.

The region-based models can obtain more accurate results than those of edge-based models by exploiting region descriptors to guide the motion of the evolving contour/surface. Chan and Vese [7] simplified the Mumford-Shah function [18] and proposed a piecewise constant (PC) model, which presumes that image intensities are statistically homogeneous in each disjoint region. However, brain MR images are usually suffered from various artifacts such as noise and intensity inhomogeneity, which makes the PC model hard to find satisfied results. In order to reduce the effect of intensity inhomogeneity, Li et al. [4] proposed a local binary fitting (LBF) energy, which assumes that image intensities are statistically homogeneous only in local regions. The LBF method can reduce the effect of intensity inhomogeneity, however, it only uses the local mean intensity of each local region, which makes the method cannot obtain accurate results when the noise is severe. In order to improve the accuracy, Wang et al. [19, 20] (Local Gaussian Distribution based method, LGD) used Gaussian distribution to fit the intensity distribution in each local region. The LGD can reduce the effect of Gaussian noise; however, if the noise is not Gaussian, it cannot obtain accurate results without any spatial information. In order to reduce the effect of noise, many researchers utilize the Markov random fields (MRF) to model the spatial correlation between neighboring pixels/voxels [21–24]. Based on this idea, Shahvaran et al. [15] utilizes the MRF to improve the robustness of LGD. Nonlocal theory is another popular method to reduce the effect of noise [25, 26]. Following this idea, Wang et al. [17] proposed a patch based LGD method (PLGD) to improve the robustness of LGD. Zhang et al. [27] found that variances in the LGD are easily affected by the intensity of the neighbor region and improved them to be piecewise constant. The method is more stable; however, it uses constant kernel to indicate the local region, which makes the method easily loses details.

A number of investigators have proved that the intensity inhomogeneity in brain MR images is harder to deal with than that of noise for segmentation methods [28–38]. The intensity inhomogeneity arises from the imperfections of the image acquisition process and manifests it-self as a smooth intensity variation across the image. Then, the intensity varies with the location of the same tissue. The intensity inhomogeneity has little effect on visual perception because the human visual system can correct it automatically. However, it changes the intensity distribution and overlaps the intensity components among different tissues, which makes the segmentation method hard to find accurate results. In order to reduce the effect of intensity inhomogeneity, Li et al. [37] improved the LBF by taking into account the bias field, which can estimate the bias field when segmenting the image. Wang et al. [13] improve d the LGD in the same way to estimate the bias field. In these methods, the smoothness of the bias field derived from the proposed energies is naturally ensured by using the Gaussian convolution in the data terms, without needing any other explicit smoothing term on the bias field. They found that when the intensity inhomogeneity level was high, an accurate bias field was still difficult to estimate. In order to obtain the estimated bias field, Li et al. [31–33] used a linear combination of orthogonal basis functions to fit the bias field, which resulted in the estimated bias field being smooth and accurate. However, these methods are based on clustering methods and have not considered spatial information, which makes the segmentation results be inaccurate.

In this paper, we propose a novel region-based level set model for brain MR image segmentation and bias correction. To integrate the spatial information, we propose a novel treatment of Hidden Markov Random field to construct the data term. Since the Student's t-distribution has one more parameter than that of Gaussian distribution, the proposed method uses the multivariate Student's t-distribution to fit the intensity distribution. In order to further reduce the effect of noise, novel anisotropic spatial information is proposed by incorporating the inner relationships among the neighborhood pixels. The anisotropic spatial information can preserve more details of the edges and corners when it is utilized into the multivariate Student's t-distribution. Following the idea of [31–33], we estimate the bias field by using a linear combination of orthogonal basis functions, which can guarantee bias field smooth, and introduce it to the data term, which makes the proposed method estimate the bias field meanwhile segmenting images. In order to make the framework more robust, we reconstructed the energy function to be convex and calculated it by using the Split Bregman theory [34, 35, 39], which allows our framework for random initialization and thereby allowing fully automated applications. The proposed method has been compared to other state-of-the-art segmentation methods in both synthetic and clinical brain MR images to show that our method can obtain results that are more accurate.

## Materials and methods

### 2.1 Materials

The synthetic brain MR images are generated from the Brain Web (http://brainweb.bic.mni.mcgill.ca/). The Brain Web provides full 3-D simulated brain data sets with three modalities: T1, T2 and PD; and can produce brain data sets with a variety of slice thicknesses, noise levels and intensity inhomogeneity levels; and provides the ground truths. The clinical brain MR images are downloaded from the Internet Brain Segmentation Repository (IBSR, http://www.cma.mgh.harvard.edu/ibsr/ which can provide full 3-D clinical brain MR image data sets and segmentation results to permit a standardized mechanism for evaluation of the sensitivity of a given analysis method for signal to noise ratio, contrast to noise ratio, shape complexity, degree of partial volume effect, etc.

### 2.2 Proposed method

#### 2.2.1 Hidden markov random field models

We consider an alphabet K={1,2,⋯,K}. Let S={1,2,⋯,N} be the set of indexes, *H* be a random field, whose state space is K, so far for ∀x∈S we have H(x)∈K. Let H be the set of all possible configurations of *H* so that $H=\Pi_{x=1}^{N}H(x)=K^{S}$. For $H{\left(x\right)}_{x\in S}\in H$ on the product space, the positive probability distribution *p*(*H*(*x*)) satisfies: p(H(x))>0,∀H(x)∈H. Let N={N(x):x∈S} be a neighborhood system on S, such as x∉N(x) and y∈N(x) if and only if x∈N(y) for ∀x,y∈S. Then the neighborhood system N can be introduced by using MRF into the previously considered random field:

p(h(x)|h(S−{x}))=p(h(x)|h(N(x))) (1)

According to the Hammersley-Clifford theorem, the MRF can be characterized by a Gibbs distribution:

$$p\left(h(x)\right)=\frac{\exp\left(U\left(h\left(x\right)\right)\right)}{Z}$$ (2)

Where *Z* is a normalizing constant given by

$$Z=\sum_{h\left(x\right)\in H}\exp\left(U\left(h\left(x\right)\right)\right)$$ (3)

and *U*(*x*) is the energy function with the form

$$U\left(h\right)=\sum_{c\in C}V_{c}\left(h\right)$$ (4)

*C* is a class of subsets of the indexes that are all neighbors, *V*_*c*_ is the clique potential associated with the clique *c* and depends on the local configuration of *c*.

Let *I* be an observable random field with state spaces I, which is also indexed by the supposed set of sites S and is given as: $I=\prod_{x=1}^{N}I(x),\;I(x)=\left\{I(x)\in R^{w}\right\}$. Given any H∈H, the random variables *I* are independent:

$$p\left(I|H\right)=\prod_{x\in S}p\left(I\left(x\right)|H\left(x\right)\right)$$ (5)

Then, the joint probability of (*I*,*H*) can be written as:

p(I,H)=p(I|H)p(H) (6)

Given the neighborhood configuration H(N(x)) of *H*(*x*), the joint probability of any pair of (*I*(*x*),*H*(*x*)) can be written by using the local characteristics of MRF [40,41] as:

p(I(x),H(x)|N(x))=p(I(x)|H(x))p(H(x)|H(N(x))) (7)

Then the marginal probability distribution of *I*(*x*), Θ, and N(x) can be written as:

$$p\left(I\left(x\right)|N\left(x\right),\Theta \right)=\sum_{k\in K}p\left(I\left(x\right);\Theta \right)p\left(k|H\left(N\left(x\right)\right)\right)$$ (8)

Where Θ is the set of parameters and in this case Θ is treated as a random variable. Compared with MRF, which is only defined with respect to *H*, HMRF is defined with respect to the pair of random variable families (*I*,*H*).

#### 2.2.2 The level set method

The snake model evolves a curve/contour from an initial position in the direction normal to the boundary of the object. One limitation of the original snake model is the explicit representation of the curve, thus topological changes (such as merging and breaking of the curve) may be hard to handle. In order to address this problem, a level set model was introduced in [42].

Later, Chan and Vese introduced a level set model (PC model) [7], which is based on the general Mumford-Shah formulation [18], for active contour segmentation. For two-phase segmentation, the minimization in [7] is defined as:

$$\begin{array}{l} E\left(c_{1},c_{2},\phi \right)=\int_{\Omega }{\left|I\left(x\right)-c_{1}\right|}^{2}H\left(\phi \right)dx+\int_{\Omega }{\left|I\left(x\right)-c_{2}\right|}^{2}\left(1-H\left(\phi \right)\right)dx\\ \;+\nu \int_{\Omega }H\left(\phi \right)dx+\mu \int_{\Omega }\nabla \left|H\left(\phi \right)\right|dx \end{array}$$ (9)

where *ϕ* is the level set function satisfying:

$$\{\begin{array}{cc} \phi \left(x\right)>0 & \mathrm{if}\;x\;\mathrm{is}\;\mathrm{inside}\;\mathrm{of}\;C\\ \phi \left(x\right)=0 & \mathrm{if}\;x\;\mathrm{is}\;\mathrm{at}\;C\\ \phi \left(x\right)<0 & \mathrm{if}\;x\;\mathrm{is}\;\mathrm{outside}\;\mathrm{of}\;C \end{array}$$ (10)

*C* is the contour, *H* is the Heaviside function: $H(x)=\frac{1}{2}\left[1+\frac{2}{\pi }\arctan\left(\frac{x}{\varepsilon }\right)\right]$. The derivative of *H* is the smoothed Dirac delta function $\delta (x)=\frac{1}{\pi }\frac{\varepsilon }{\varepsilon^{2}+x^{2}}$ [36]. *ν* and *μ* are the weighting parameters. $\int_{\Omega }H(\phi )dx$ is the area term and $\int_{\Omega }\nabla \left|H(\phi )\right|dx$ is the length term.

In order to segment images into more regions, Vese and Chan [43] improved PC model into multiphase model. For segmenting brain MR images into four classes: WM, GM, CSF and the background, the improved PC model can be written as:

$$\begin{array}{l} E\left(c_{1},c_{2},c_{3},c_{4},\phi_{1},\phi_{2}\right)=\sum_{k=1}^{K}\int_{\Omega }{\left|I\left(x\right)-c_{k}\right|}^{2}M_{k}\left(\phi_{1},\phi_{2}\right)dx\\ \;+\nu \int_{\Omega }\nabla \left|H\left(\phi_{1}\right)\right|dx+\nu \int_{\Omega }\nabla \left|H\left(\phi_{2}\right)\right|dx \end{array}$$ (11)

Where *M*_*i*_(*ϕ*_1_, *ϕ*_2_) are functions of *ϕ*, which are designed such that $\sum_{i=1}^{N}M_{i}\left(\phi_{1},\phi_{2}\right)=1$. In this paper: *M*_1_(*ϕ*) = *H*(*ϕ*_1_)*H*(*ϕ*_2_), *M*_2_(*ϕ*) = *H*(*ϕ*_1_)(1−*H*(*ϕ*_2_)), *M*_3_(*ϕ*) = (1−*H*(*ϕ*_1_))*H*(*ϕ*_2_), *M*_4_(*ϕ*) = (1−*H*(*ϕ*_1_))(1−*H*(*ϕ*_2_)).

The improved PC model assumes that image intensities are statistically homogeneous in each disjoint region, which makes it sensitive to intensity inhomogeneity. To deal with the effect of intensity inhomogeneity, the LGD method used the Gaussian distribution to describe local region information and the energy function is defined as follows:

$$\begin{array}{l} E\left(\phi ,\Theta \right)=-\lambda_{1}\int_{\Omega }\int_{\Omega_{1}}\omega \left(x-y\right)\log p_{1,y}\left(I\left(x\right)\right)M_{1}\left(\phi \left(x\right)\right)dxdy\\ \;-\lambda_{2}\int_{\Omega }\int_{\Omega_{2}}\omega \left(x-y\right)\log p_{2,y}\left(I\left(x\right)\right)M_{2}\left(\phi \left(x\right)\right)dxdy\\ \;-\lambda_{3}\int_{\Omega }\int_{\Omega_{3}}\omega \left(x-y\right)\log p_{3,y}\left(I\left(x\right)\right)M_{3}\left(\phi \left(x\right)\right)dxdy\\ \;-\lambda_{4}\int_{\Omega }\int_{\Omega_{4}}\omega \left(x-y\right)\log p_{4,y}\left(I\left(x\right)\right)M_{4}\left(\phi \left(x\right)\right)dxdy\\ \;+\nu \int \left|\nabla H\left(\phi_{1}\right)\right|dy+\nu \int \left|\nabla H\left(\phi_{2}\right)\right|dy\\ \;+\mu \int \frac{1}{2}{\left(\left|\nabla \phi_{1}\right|-1\right)}^{2}dy+\mu \int \frac{1}{2}{\left(\left|\nabla \phi_{2}\right|-1\right)}^{2}dy \end{array}$$ (12)

Where *Θ* is the parameter of Gaussian distribution, *ω*(*x*−*y*) is a non-negative weighting function such that *ω*(*x*−*y*) = 0 for |*x*−*y*| > *r* and $\int_{\Lambda_{y}}\omega \left(x-y\right)dx=1$. *r* is the radius of local region. In the method, the weighting function is chosen as a Gaussian kernel. $p_{i,y}\left(I\left(x\right)\right)=\frac{1}{\sqrt{2\pi }\sigma_{i,y}}\exp\left(-\frac{{\left(I(x)-u_{i}(y)\right)}^{2}}{2\sigma_{i,y}^{2}}\right),\;\lambda_{i},\;i=1,2,3,4$, *ν* and *μ* are nonnegative constant. ∫∇|H(ϕ)|dy serves to regularize the zero level contour of *ϕ*, while $\int \frac{1}{2}{\left(\left|\nabla \phi \right|-1\right)}^{2}dy$ regularizes the entire level set function *ϕ* by penalizing its deviation from signed distance[4]. The LGD method can reduce the effect of Gaussian noise by using Gaussian distribution; however, when the images have strong noise and severe intensity inhomogeneity, the method is hard to obtain accurate results. In order to reduce the effect of intensity inhomogeneity, our previous work [38] introduced the bias field into LGD:

$$\begin{array}{l} E\left(\phi ,\Theta \right)=-\lambda_{1}\int_{\Omega }\int_{\Omega_{1}}\omega \left(x-y\right)\log p_{1,y}\left(\tilde{I}\left(x\right)-\tilde{B}\left(y\right)\right)M_{1}\left(\phi \left(x\right)\right)dxdy\\ \;-\lambda_{2}\int_{\Omega }\int_{\Omega_{2}}\omega \left(x-y\right)\log p_{2,y}\left(\tilde{I}\left(x\right)-\tilde{B}\left(y\right)\right)M_{2}\left(\phi \left(x\right)\right)dxdy\\ \;-\lambda_{3}\int_{\Omega }\int_{\Omega_{3}}\omega \left(x-y\right)\log p_{3,y}\left(\tilde{I}\left(x\right)-\tilde{B}\left(y\right)\right)M_{3}\left(\phi \left(x\right)\right)dxdy\\ \;-\lambda_{4}\int_{\Omega }\int_{\Omega_{4}}\omega \left(x-y\right)\log p_{4,y}\left(\tilde{I}\left(x\right)-\tilde{B}\left(y\right)\right)M_{4}\left(\phi \left(x\right)\right)dxdy\\ \;+\nu \int \left|\nabla H\left(\phi_{1}\right)\right|dy+\nu \int \left|\nabla H\left(\phi_{2}\right)\right|dy\\ \;+\mu \int \frac{1}{2}{\left(\left|\nabla \phi_{1}\right|-1\right)}^{2}dy+\mu \int \frac{1}{2}{\left(\left|\nabla \phi_{2}\right|-1\right)}^{2}dy \end{array}$$ (13)

Where $\tilde{I}=log\left(I\right),\;\tilde{B}=log\left(B\right)$. *B* is the bias field, which satisfies: log(*I*) = log((*J* + *n*)⋅*B*) = log(*J* + *n*) + log(*B*). *J* is true signal, *n* is noise. The proposed method can estimate a bias field when segmenting images, however, as analyzed above, the method still sensitive to noise without any spatial information.

#### 2.2.3 Anisotropic spatial information

Baudes et al. [44] have proved that a non-local neighbor patch contains more information than that of signal pixel in image. Follows this idea, many proposed methods [45–47] used the non-local neighbor patch information to reduce the effect of noise. In non-local neighbor patch information based methods, the distance between patches is defined as:

$$d\left(P_{x},P_{y}\right)=\sum_{i=1}^{\left|P\left(x\right)\right|}{\left(I\left(P_{x,i}\right)-I\left(P_{y,i}\right)\right)}^{2}$$ (14)

**Fig 1:**
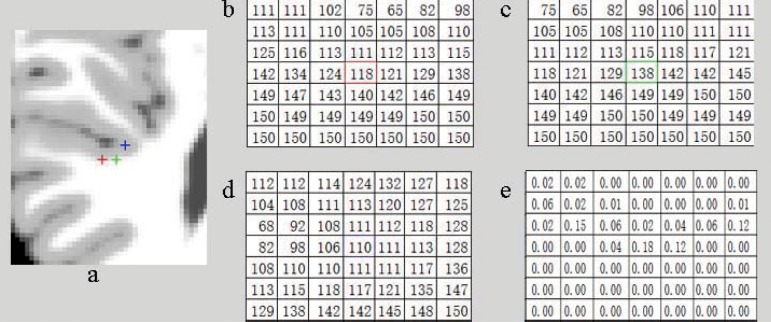
Analysis of the neighborhood similarities. (a) Initial image; (b-d) are the intensities information of points marked with red plus, green plus and blue plus, respectively; (e) the inner-relationship in (b).

Where *P*_*x*_ and *P*_*y*_ are the neighbor patches centered at *x* and *y*, respectively. |*P*(*x*)| is the total number of pixels in the neighbor patch. From Eq (14), we can find that each pixel in the patch has same weight, which makes the patch information be isotropic. Fig 1(A) shows a part of simulated brain MR image. The points, marked with red plus (set as A), green plus (set as B) and blue plus (set as C), belong to GM, WM and GM, respectively. Fig 1B–1D shows the intensities information of the square neighbor patches centered at the corresponding pixels with size 7 × 7. By using Eq (14), the distance between *P*_*A*_ and *P*_*B*_ is 97.73, and the distance between *P*_*A*_ and *P*_*C*_ is 182.84, means that that the point *B* is more similar to *A* than that of *C*. However, the point *B* belongs to WM, which makes that the neighbor patch based method hard to find accurate results. In order to deal with this problem, we proposed an anisotropic neighbor patch inner-relationship:

$$R_{x}\left(y\right)=\{\begin{array}{cc} \frac{e^{-\beta {\left\|I^{\prime }\left(x\right)-I\left(y\right)\right\|}_{2}^{2}}}{\int_{y\in \left\{P_{x}\right\}}e^{-\beta {\left\|I^{\prime }\left(x\right)-I\left(y\right)\right\|}_{2}^{2}}dy} & \mathrm{for}\;x\in \left\{\mathrm{Sole}\;\mathrm{points}\right\}\\ \frac{e^{-\beta {\left\|I\left(x\right)-I\left(y\right)\right\|}_{2}^{2}}}{\int_{y\in \left\{P_{x}\right\}}e^{-\beta {\left\|I\left(x\right)-I\left(y\right)\right\|}_{2}^{2}}dy} & \mathrm{otherwise} \end{array}$$ (15)

where *I*′(*x*) is the mean intensities of *P*_*x*_ without *x*. *β* is a nonnegative constant, which is dependent on the standard deviation of the image noise *σ*, which can be estimated by using the method in [48]. From Eq (15), it can be found that the pixels in the neighbor patch with similar intensities to the center pixel will have higher weights, which make the inner-relationship based neighbor patch be anisotropic and contains more details.

Due to the effect of the noise, the intensities of some pixels are much larger or less than all other pixels in the neighbor patch and we refer to these pixels as sole points. For a pixel *x*, if $\left(1/\int_{y\in P_{x}}e^{-\beta \left({\left\|I\left(x\right)-I\left(y\right)\right\|}^{2}\right)}dy\right)>T$, we regard *x* as a sole point. *T* is non-negative constant (the default value is 0.75 in this paper). The distance, calculated by using $d\left(P_{x},P_{y}\right)=\sum_{i=1}^{\left|P(x)\right|}{\left(R_{x,i}\left(I\left(P_{x,i}\right)-I\left(P_{y,i}\right)\right)\right)}^{2}$, between *P*_*A*_ and *P*_*B*_ is 4.73, and between *P*_*A*_ and *P*_*C*_ is 4.68, which means that the point *C* is more similar to *A* than that of *B*. From the definition in Eq (15), it can be found that the neighbor patch is anisotropic, which can contain more detail information.

#### 2.2.4 Level set-type treatment of the HMRF model

As analyzed in our previous work [38], the energy function (Eq 13) is non-convex, which makes the method easily trap into a local minimum. In order to obtain global mini-mum, we improved the energy function by using the Split Bregman method [49], which is a technique for solving a variety of L1-regularized optimization problems, and is particularly effective for problems involving total-variation regularization. In this section, we use two segmentation variables *u*_1_ ∈ [0,1] and *u*_2_ ∈ [0,1] to represent the membership functions of four regions: *M*_1_(*u*_1_,*u*_2_) = *u*_1_*u*_2_, *M*_2_(*u*_1_,*u*_2_) = *u*_1_(1−*u*_2_), *M*_3_(*u*_1_,*u*_2_) = (1−*u*_1_)*u*_2_ and *M*_4_(*u*_1_,*u*_2_) = (1−*u*_1_)(1−*u*_2_). The HMRF based energy function can be written as:

$$\begin{array}{l} E\left(u_{1},u_{2},\Theta \right)=\sum_{k=1}^{4}\int -\log\left(p\left(I\left(x\right),H\left(x\right)=k\right)\right)M_{i}\left(x\right)dx+\nu \int \left|\nabla u_{1}\right|dx+\nu \int \left|\nabla u_{2}\right|dx\\ \;=\sum_{k=1}^{4}\int -\log\left(p\left(I\left(x\right)|H\left(x\right)=k\right)p\left(H\left(x\right)=k|H\left(N\left(x\right)\right)\right)\right)M_{i}\left(x\right)dx\\ \;+\nu \int \left|\nabla u_{1}\right|dx+\nu \int \left|\nabla u_{2}\right|dx \end{array}$$ (16)

When the observed data follows the Gaussian distribution, the Gaussian function can fit the distribution accurately. However, in brain MR images, the intensities are not always following the Gaussian distribution, which makes Gaussian distribution based methods hard to find satisfied results. By way of contrast, the Student's t-distribution [50] has one more parameter, named as degree of freedom *υ*. When *υ* is set as 1, the Student's t-distribution reduces to be the Cauchy distribution. The Student's t-distribution becomes closer to the Gaussian distribution as *υ* increases. Hence, Student's t-distribution can model the observed data more powerfully and flexibly than Gaussian distribution. Then, *p*(*I*(*x*)|*H*(*x*) = *k*) can be defined by using multivariate Student's t-distribution:

$$p\left(I\left(x\right)|H\left(x\right)=k\right)=\frac{\Gamma \left(\frac{\upsilon_{k}+D}{2}\right){\left|\Sigma_{k}\right|}^{-1/2}}{\Gamma \left(\frac{\upsilon_{k}}{2}\right){\left(\pi \upsilon_{k}\right)}^{\left(D/2\right)}}{\left[1+\frac{{\left(I\left(x\right)-\mu_{k}\right)}^{T}\Sigma_{k}^{-1}\left(I\left(x\right)-\mu_{k}\right)}{\upsilon_{k}}\right]}^{-\frac{\upsilon_{k}+D}{2}}$$ (17)

where *μ*, Σ and *υ* are mean vector, covariance matrix and degree of freedom, respectively. In order to improve the robustness, we introduce the anisotropic spatial information into the energy function:

$$\begin{array}{l} E\left(u_{1},u_{2},\mu ,\Sigma ,v\right)\\ =\sum_{k=1}^{4}\int -\log p\left(I\left(P_{x}\right),H\left(x\right)=k\right)M_{i}\left(x\right)dx+\nu \int \left|\nabla u_{1}\right|dx+\nu \int \left|\nabla u_{2}\right|dx\\ =\sum_{k=1}^{4}\int -\log\left(p\left(I\left(x\right)|H\left(x\right)=k\right)p\left(H\left(x\right)=k|H\left(N\left(x\right)\right)\right)\right)M_{i}\left(x\right)dx\\ +\nu \int \left|\nabla u_{1}\right|dx+\nu \int \left|\nabla u_{2}\right|dx\\ =\sum_{k=1}^{4}\int -\log\left(\begin{array}{l} \left(\frac{\Gamma \left(\frac{\upsilon_{k}+D}{2}\right){\left|\Sigma_{k}\right|}^{-1/2}}{\Gamma \left(\frac{\upsilon_{k}}{2}\right){\left(\pi \upsilon_{k}\right)}^{\left(D/2\right)}}{\left[1+\frac{{\left(I\left(x\right)-\mu_{k}\right)}^{T}\Sigma_{k}^{-1}\left(I\left(x\right)-\mu_{k}\right)}{\upsilon_{k}}\right]}^{-\frac{\upsilon_{k}+D}{2}}\right)\\ p\left(H\left(x\right)=k|H\left(N\left(x\right)\right)\right) \end{array}\right)M_{i}\left(x\right)dx\\ +\nu \int \left|\nabla u_{1}\right|dx+\nu \int \left|\nabla u_{2}\right|dx \end{array}$$ (18)

where $P_{x}^{\prime }$ and $R_{x}^{\prime }$ are vectors generated from the neighbor patch *P*_*x*_ and the inner-relationship *R*_*x*_, respectively. *b* is the bias field.

In order to obtain a smooth bias field, Li et al. [31–33] model the bias field to be a linear combination of L smooth basis functions *s*_1_,*s*_1_⋯*s*_*L*_:

$$b\left(x\right)=\sum_{l=1}^{L}q_{l}s_{l}\left(x\right)$$ (19)

Where *q*_*l*_ ∈ *R*, *l* = 1,⋯,*L*, are the combination coefficients. Orthogonal polynomials are usually used as the basis functions, which satisfy:

$$\int_{\Omega }s_{i}\left(x\right)s_{j}\left(x\right)dx=\delta_{i,j}$$ (20)

Where *δ*_*i*,*j*_ = 1 for *i* = *j* and *δ*_*i*,*j*_ = 0 for *i* ≠ *j*. Following the idea shown in [31–33], we use the Legendre orthogonal polynomials as the basis functions. In 2D case, the size of the parameter is *L* = (*m* + 1)(*m* + 2)/2, where *m* is the degree of the Legendre polynomials. In 3D case, the size of the parameter is *L* = (*m* + 1)(*m* + 2)(*m* + 3)/6. The choice of *m* depends on prior knowledge of the coil and the expected type. To simplify, the bias field can be written as:

$$b\left(x\right)=Q^{T}S\left(x\right)$$ (21)

Where *Q* = (*q*_1_,*q*_2_,⋯,*q*_*L*_)^*T*^, S(*x*) = (*s*_1_(*x*),*s*_2_(*x*),⋯,*s*_*L*_(*x*))^*T*^.

Remark 1: Although the local patch information has been used to improve segmentation methods to reduce the effect of the noise; however, most of the methods use isotropic neighbor patch information, which makes them easily lose details [51]. Our method can preserve more detail information by using anisotropic neighbor patch information. Furthermore, we use HMRF to model the spatial correlation between neighboring pixels/voxels and further improve the robustness of our method.

Remark 2: Compared to the method in [13], we use a multivariate Student's t-distribution to fit the intensity distribution of each region in the image, which can improve the ability to identify the class for each pixel. In our method, we use the local neighbor patch information to reduce the effect of noise, which also makes our method can only use one integration, instead two integrations in the LBF [4] and LGD [19,20], to construct the data term. Our method is more efficient than LBF and LGD.

Remark 3: In LBF and LGD, the smoothness of the bias field is ensured by using a Gaussian convolution; however, the parameter of the Gaussian convolution needs to be changed when the methods segmenting different images. In our method, we use orthogonal polynomials as the basis functions, which makes our method does not need any Gaussian convolution and can estimate the bias field, even when the intensity inhomogeneity is severe.

Remark 4: Although Student's t-distribution has been widely used in segmentation methods [48] and obtained more accurate results than Gaussian distribution based methods. However, the Student's t-distribution is sensitive to noise. Furthermore, the Student's t-distribution based methods are sensitive to initialization. In our method, we use the anisotropic patch information to improve the robustness of the Student's t-distribution on noise. Furthermore, in our method, the energy function can be rewritten as convex and calculated by using Split Bregman Method, which makes our method can obtain accurate results with randomly initialization.

Remark 5: In our method, the bias field is modeled by using Legendre orthogonal polynomials, which can be found in [31–33]. The methods in [31–33] are based on clustering theories and have not considering spatial information, which makes the segmentation results be inaccurate and reduce the accuracy of the estimated bias field. Our method uses the anisotropic information and Student's t-distribution to model local information to improve the accuracy of the segmentation results and makes the estimated bias field more accurate.

#### 2.2.5 Split Bregman method for minimization of energy

The split Bregman technique is used to minimize the energy function in a more efficient way and obtain the global minima. The proposed model thus can improve the robustness and efficiency, while inheriting the desirable ability to estimate the bias field when segmenting images.

When given *Θ* and *b*, we minimize *E*(*u*_1_,*u*_2_,*Θ*,*b*) with respect to *u*_1_ using the gradient descent method by solving the gradient flow equation as:

$$\frac{\partial u_{1}}{\partial t}=-\delta (u_{1})(-e_{1}H(u_{2})-e_{2}(1-H(u_{2}))+e_{3}H(u_{2})+e_{4}(1-H(u_{2}))+\nu \delta (u_{1})div\left(\frac{\nabla u_{1}}{\left|\nabla u_{1}\right|}\right))$$ (22)

Where *e*_*i*_(*x*) = −log *p*(*I*(*P*_*x*_),*H*(*x*) = *i*)

In the same manner, minimizing the energy functional *E* with respect to *u*_2_, we derive the gradient descent flow:

$$\frac{\partial u_{2}}{\partial t}=-\delta (u_{2})(-e_{1}H(u_{1})+e_{2}H(u_{1})-e_{3}(1-H(u_{1}))+e_{4}(1-H(u_{1}))+\nu \delta (u_{1})div\left(\frac{\nabla u_{1}}{\left|\nabla u_{1}\right|}\right))$$ (23)

Without loss of generality, we take *ν* = 1 and the stationary solution of (22) and (23) coincides with the stationary solution of [38]:

$$\frac{\partial u_{1}}{\partial t}=-(-e_{1}H(u_{2})-e_{2}(1-H(u_{2}))+e_{3}H(u_{2})+e_{4}(1-H(u_{2}))+\nu \delta (u_{1})div\left(\frac{\nabla u_{1}}{\left|\nabla u_{1}\right|}\right))$$ (24)

$$\frac{\partial u_{2}}{\partial t}=-(-e_{1}H(u_{1})+e_{2}H(u_{1})-e_{3}(1-H(u_{1}))+e_{4}(1-H(u_{1}))+\nu \delta (u_{1})div\left(\frac{\nabla u_{1}}{\left|\nabla u_{1}\right|}\right))$$ (25)

The simplified flow represents the gradient descent for minimizing the energy:

$$\left\{ \begin{array}{l} \min_{u_{1}\in \left[0,1\right],\Theta ,b}E_{1}\left(u_{1},\Theta ,b\right)=\nu \int \left|\nabla u_{1}\right|dx+\int u_{1}\left(x\right)e_{11}\left(x\right)dx+\int \left(1-u_{1}\left(x\right)\right)e_{12}\left(x\right)dx\\ \min_{u_{2}\in \left[0,1\right],\Theta ,b}E_{2}\left(u_{2},\Theta ,b\right)=\nu \int \left|\nabla u_{2}\right|dx+\int u_{2}\left(x\right)e_{21}\left(x\right)dx+\int \left(1-u_{2}\left(x\right)\right)e_{22}\left(x\right)dx \end{array}\right.$$ (26)

Where

$$\begin{array}{l} e_{11}\left(x\right)=\left(-\log\left(p\left(I\left(P_{x}\right),H\left(x\right)=1\right)\right)u_{2}\left(x\right)-\log\left(p\left(I\left(P_{x}\right),H\left(x\right)=2\right)\right)\left(1-u_{2}\left(x\right)\right)\right)\\ e_{12}\left(x\right)=\left(-\log\left(p\left(I\left(P_{x}\right),H\left(x\right)=3\right)\right)u_{2}\left(x\right)-\log\left(p\left(I\left(P_{x}\right),H\left(x\right)=4\right)\right)\left(1-u_{2}\left(x\right)\right)\right)\\ e_{21}\left(x\right)=\left(-\log\left(p\left(I\left(P_{x}\right),H\left(x\right)=1\right)\right)u_{1}\left(x\right)-\log\left(p\left(I\left(P_{x}\right),H\left(x\right)=3\right)\right)\left(1-u_{1}\left(x\right)\right)\right)\\ e_{22}\left(x\right)=\left(-\log\left(p\left(I\left(P_{x}\right),H\left(x\right)=2\right)\right)u_{1}\left(x\right)-\log\left(p\left(I\left(P_{x}\right),H\left(x\right)=4\right)\right)\left(1-u_{1}\left(x\right)\right)\right) \end{array}.$$

It has been proved [52] that when *u*_2_ is fixed, if ${\hat{u}}_{1}$ is any minimizer of *E*_1_, for ∀T∈(0,1) we have that the characteristic function

$$1_{\Omega_{C\left(T\right)}}=\left\{y|{\hat{u}}_{1}\left(y\right)>T\right\}$$

where *C* is the boundary of the set Ω_*C*_, is a global minimization of *E*_1_. It can also be proved that for fixed *u*_1_, the characteristic function $1_{\Omega_{C\left(T\right)}}=\left\{y|{\hat{u}}_{2}\left(y\right)>T\right\}$ is a global minimization of *E*_2_.

Following the idea of [52], we introduce a new vectorial function *di* = ∇*u*_*i*_ and rewrite Eq (26) as:

$$\{\begin{array}{cc} \underset{u_{1}\in \left[0,1\right],d_{1}}{\min}{\tilde{E}}_{1}\left(u_{1},\Theta ,d_{1}\right)=\nu \int \left(\left|d_{1}\right|+u_{1}e_{11}+\left(1-u_{1}\right)e_{12}\right)dy, & \mathrm{such}\;\mathrm{that}\;d_{1}=\nabla u_{1}\\ \underset{u_{2}\in \left[0,1\right],d_{2}}{\min}{\tilde{E}}_{2}\left(u_{2},\Theta ,d_{2}\right)=\nu \int \left(\left|d_{2}\right|+u_{2}e_{21}+\left(1-u_{2}\right)e_{22}\right)dy, & \mathrm{such}\;\mathrm{that}\;d_{2}=\nabla u_{2} \end{array}$$ (27)

For simplicity, we only consider the minimization of the energy ${\tilde{E}}_{1}$, while the minimization of the energy ${\tilde{E}}_{2}$ can be solved in the same manner. Adding a new vector *p*_1_←∇*u*_1_ into a quadratic penalty function, we obtain the following optimization function:

$$\left\{ \begin{array}{l} \left(u_{1}^{t+1},d_{1}^{t+1}\right)=\underset{1\leq u_{1}\leq 1,d_{1}}{\arg\;\min}\nu \int \left(\left|d_{1}\right|+u_{1}e_{11}+\left(1-u_{1}\right)e_{12}+\frac{\gamma }{2}{\left\|d_{1}-\nabla u_{1}-p_{1}^{t}\right\|}^{2}\right)dy,\\ p_{1}^{t+1}=p_{1}^{t}+\nabla u_{1}^{t+1}-d_{1}^{t+1} \end{array}\right.$$ (28)

For the fixed *d*_1_, we can derive the Euler-Lagrange equation of the optimization problem Eq (28) with respect to *u*_1_:

$$\Delta u_{1}=\frac{1}{\gamma }\left(e_{11}-e_{12}\right)-div\left(p-d\right),\;u_{1}\in \left[0,1\right]$$ (29)

Where Δ is the Laplacian operator.

In 3D case, by using central discretization for the Laplacian operator and backward difference for the divergence operator, a fast approximated solution for Eq (28) is:

$${\left(u_{1}\right)}_{i,j,k}=\max\left\{\min\left\{\alpha_{i,j,k},1\right\},0\right\}$$ (30)

where (*i*, *j*, *k*) is the position in image coordinate and

$$\alpha_{i,j,k}=\frac{1}{6}\left[{\left(u_{1}\right)}_{i-1,j,k}+{\left(u_{1}\right)}_{i+1,j,k}+{\left(u_{1}\right)}_{i,j-1,k}+{\left(u_{1}\right)}_{i,j+1,k}+{\left(u_{1}\right)}_{i,j,k-1}+{\left(u_{1}\right)}_{i,j,k+1}-\frac{1}{\gamma }{\left(e_{11}-e_{12}\right)}_{i,j,k}+\beta_{i,j,k}\right],$$

$$\beta_{i,j,k}=d_{i-1,j,k}^{x}-d_{i,j,k}^{x}-p_{i-1,j,k}^{x}+p_{i,j,k}^{x}+d_{i,j-1,k}^{y}-d_{i-1,j,k}^{y}-p_{i,j-1,k}^{y}+p_{i,j,k}^{y}+d_{i,j,k-1}^{z}-d_{i,j,k}^{z}-p_{i,j,k-1}^{z}+p_{i,j,k}^{z}.$$

For the fixed *u*_1_, the minimization solution *d*^*t*+1^ is performed by using the following formula:

$$d^{t+1}=\frac{\nabla u_{1}^{t+1}+p^{t}}{\left\|\nabla u_{1}^{t+1}+p^{t}\right\|}\max\left\{\nabla u_{1}^{t+1}+p^{t}-\frac{\nu }{\gamma },0\right\}$$ (31)

Before update *u*_1_ and *u*_2_, we first need calculate other parameters of the energy function. For fixed *u*_1_, *u*_2_ and *b*, the parameter *μ*, Σ and *υ* can be calculated by taking the derivative of *E* with respect to *μ*, Σ and *υ* and setting the results to zero, respectively. For updating *μ*_*k*_, we have:

$$\begin{array}{l} \frac{\partial E}{\partial \mu_{k}}=\frac{\begin{array}{l} \partial \sum_{k=1}^{4}\int -\log\left(\begin{array}{l} \left(\frac{\Gamma \left(\frac{\upsilon_{k}+D}{2}\right){\left|\Sigma_{k}\right|}^{-1/2}}{\Gamma \left(\frac{\upsilon_{k}}{2}\right){\left(\pi \upsilon_{k}\right)}^{\left(D/2\right)}}{\left[1+\frac{{\left(I\left(x\right)-\mu_{k}\right)}^{T}\Sigma_{k}^{-1}\left(I\left(x\right)-\mu_{k}\right)}{\upsilon_{k}}\right]}^{-\frac{\upsilon_{k}+D}{2}}\right)\\ \cdot p\left(H\left(x\right)=k|H\left(N\left(x\right)\right)\right) \end{array}\right)M_{k}\left(x\right)dx\\ \;+\nu \int \left|\nabla u_{1}\right|dx+\nu \int \left|\nabla u_{2}\right|dx \end{array}}{\partial \mu_{k}}\\ \;=0 \end{array}$$ (32)

It is hard to calculate *μ*_*k*_ directly from Eq (32). Fortunately, it has been proved that if *I* follows the Student's t-distribution *p*(*μ*,Σ,*υ*), it can be considered following a Gaussian distribution *N*(*μ*,Σ/*t*), where *t* is a scaling factor following a Gamma-distribution [50,53] and can be calculated by:

$$t_{k}\left(x\right)=\frac{\upsilon_{k}+D}{\upsilon_{k}+\left({\left(R_{x}^{\prime }\cdot \left(I\left(P_{x}^{\prime }\right)-b\left(x\right)\mu_{k}\right)\right)}^{T}\Sigma_{k}^{-1}\left(R_{x}^{\prime }\cdot \left(I\left(P_{x}^{\prime }\right)-b\left(x\right)\mu_{k}\right)\right)\right)}$$ (33)

So *p*(*I*(*P*_*x*_)|*H*(*x*) = *k*) can be written as:

$$p(I(P_{x})|H(x)=k)=\frac{1}{\sqrt{\left(2\pi \right)}{\left|\frac{\Sigma_{k}}{t_{k}\left(x\right)}\right|}^{\frac{1}{2}}}\exp\left(\begin{array}{l} -\frac{1}{2}t_{k}\left(x\right){\left(R_{x}^{\prime }\cdot \left(I\left(P_{x}^{\prime }\right)-b\left(x\right)\mu_{k}\right)\right)}^{T}\\ \Sigma_{k}^{-1}\left(R_{x}^{\prime }\cdot \left(I\left(P_{x}^{\prime }\right)-b\left(x\right)\mu_{k}\right)\right) \end{array}\right)$$ (34)

Substituting Eq (34) into Eq (32), we can obtain:

$$\begin{array}{l} \frac{\partial E}{\partial \mu_{k}}=\frac{\begin{array}{l} \partial \sum_{k=1}^{4}\int -\log\left(\begin{array}{l} \frac{1}{\sqrt{\left(2\pi \right)}{\left|\frac{\Sigma_{k}}{t_{k}\left(x\right)}\right|}^{\frac{1}{2}}}\exp\left(\begin{array}{l} -\frac{1}{2}t_{k}\left(x\right){\left(R_{x}^{\prime }\cdot \left(I\left(P_{x}^{\prime }\right)-b\left(x\right)\mu_{k}\right)\right)}^{T}\\ \Sigma_{k}^{-1}\left(R_{x}^{\prime }\cdot \left(I\left(P_{x}^{\prime }\right)-b\left(x\right)\mu_{k}\right)\right) \end{array}\right)\\ p\left(H\left(x\right)=k|H\left(N\left(x\right)\right)\right) \end{array}\right)M_{k}\left(x\right)dx\\ \;+\nu \int \left|\nabla u_{1}\right|dx+\nu \int \left|\nabla u_{2}\right|dx \end{array}}{\partial \mu_{k}}\\ \;=\frac{\partial \sum_{k=1}^{4}\int \left(\frac{1}{2}t_{k}\left(x\right){\left(R_{x}^{\prime }\cdot \left(I\left(P_{x}^{\prime }\right)-b\left(x\right)\mu_{k}\right)\right)}^{T}\Sigma_{k}^{-1}\left(R_{x}^{\prime }\cdot \left(I\left(P_{x}^{\prime }\right)-b\left(x\right)\mu_{k}\right)\right)\right)M_{k}\left(x\right)dx}{\partial \mu_{k}}\\ \;=0 \end{array}$$ (35)

Then, we can obtain:

$$\mu_{k}=\int t_{k}\left(x\right)b\left(x\right)M_{k}\left(x\right)I\left(P_{x}^{\prime }\right)dx./\int t_{k}\left(x\right)b^{2}\left(x\right)M_{k}\left(x\right)dx$$ (36)

Where ./ is point division. Similarly, we calculate Σ_*k*_ as:

$$\Sigma_{k}=\frac{\int t_{k}\left(x\right)M_{k}\left(x\right)\left(R_{x}^{\prime }\cdot \left(I\left(P_{x}^{\prime }\right)-b\left(x\right)\mu_{k}\right)\right){\left(R_{x}^{\prime }\cdot \left(I\left(P_{x}^{\prime }\right)-b\left(x\right)\mu_{k}\right)\right)}^{T}dx}{\int M_{k}\left(x\right)dx}$$ (37)

Setting the partial derivative of *E* with respect to *υ* and setting the results to zero, we have

$$\ln\left(\frac{\upsilon_{k}}{2}\right)-\psi \left(\frac{\upsilon_{k}}{2}\right)+1+\frac{\int \left(\ln\left(t_{k}\left(x\right)\right)-t_{k}\left(x\right)\right)M_{k}\left(x\right)dx}{\int M_{k}\left(x\right)dx}+\psi \left(\frac{\upsilon_{k}+D}{2}\right)-\ln\left(\frac{\upsilon_{k}}{2}\right)=0$$ (38)

where $\psi \left(x\right)=\frac{d}{dx}\ln\Gamma (x)$.

For fixed *M*, *μ*, Σ and *υ*, taking the derivative of *E* with respect to *Q* and setting the result to zero, we have:

$$\begin{array}{l} \frac{\partial E}{\partial Q}=\frac{\begin{array}{l} \partial \sum_{k=1}^{4}\int -\log\left(\begin{array}{l} \frac{1}{\sqrt{\left(2\pi \right)}{\left|\frac{\Sigma_{k}}{t_{k}\left(x\right)}\right|}^{\frac{1}{2}}}\exp\left(\begin{array}{l} -\frac{1}{2}t_{k}\left(x\right){\left(R_{x}^{\prime }\cdot \left(I\left(P_{x}^{\prime }\right)-Q^{T}S\left(x\right)\mu_{k}\right)\right)}^{T}\\ \Sigma_{k}^{-1}\left(R_{x}^{\prime }\cdot \left(I\left(P_{x}^{\prime }\right)-Q^{T}S\left(x\right)\mu_{k}\right)\right) \end{array}\right)\\ p\left(H\left(x\right)=k|H\left(N\left(x\right)\right)\right) \end{array}\right)M_{k}\left(x\right)dx\\ \;+\nu \int \left|\nabla u_{1}\right|dx+\nu \int \left|\nabla u_{2}\right|dx \end{array}}{\partial Q}\\ \;=\frac{\partial \sum_{k=1}^{4}\int \left(\frac{1}{2}t_{k}\left(x\right){\left(R_{x}^{\prime }\cdot \left(I\left(P_{x}^{\prime }\right)-Q^{T}S\left(x\right)\mu_{k}\right)\right)}^{T}\Sigma_{k}^{-1}\left(R_{x}^{\prime }\cdot \left(I\left(P_{x}^{\prime }\right)-Q^{T}S\left(x\right)\mu_{k}\right)\right)\right)M_{k}\left(x\right)dx}{\partial Q}\\ \;=0 \end{array}$$ (39)

Then we can obtain:

$$Q=A^{-1}W$$ (40)

where $A=\int S\left(x\right)S{\left(x\right)}^{T}\sum_{k=1}^{K}M_{k}\left(x\right)t_{k}\left(x\right){\left(R_{x}^{\prime }\cdot \mu_{k}\right)}^{T}\sum_{k}^{-1}\left(R_{x}^{\prime }\cdot \mu_{k}\right)dx$ is a *L*×*L* matrix, which is inverse-able and the similar proof of the stability can be seen in [31]. $W=\int S\left(x\right)\sum_{k=1}^{K}M_{k}\left(x\right)t_{k}\left(x\right){\left(R_{x}^{\prime }\cdot I\left(P_{x}^{\prime }\right)\right)}^{T}\sum_{k}^{-1}\left(R_{x}^{\prime }\cdot \mu_{k}\right)dx$ is a *L*×1 vector.

For a deep understanding of our method, the computation process of our algorithm is summarized as follows:

Step.1 Initialize *u*_1_, *u*_2_, *Θ* and *b*. In our method, *u*_1_ and *u*_2_ can be initialized randomly and b is a matrix of ones.
Step.2 Update *Θ* by using Eqs (36), (37) and (38).
Step.3 Update *Q* by using Eq (40).
Step.4 Update *u* by using Eq (30).
Step.5 If the distance between the newly obtained level sets and old ones is less than a user-specified small threshold (in this paper, we set ε = 0.001), stop the iteration; otherwise, go to Step 2.

## Results

In this section, we apply the proposed method to segment the brain MR images into GM, WM, CSF and background. Unless otherwise specified, the parameters used in our experiments are set as follows: The *u*_1_ and *u*_2_ are initialized randomly. The size of neighbor patch is set as 3 × 3. The nonnegative constant *β* is set as 0.02. The degree of basis functions is set as *m* = 4 and hence the number of the basis functions *L* is 15. *ν* is set as 100 and *γ* is set as 1. In this paper, we conduct the image segmentation task by imposing HMRF on the image pixel labels. In our experiments, p(H(x)=k|H(N(x))) is:

$$p\left(H\left(x\right)=k|H\left(N\left(x\right)\right)\right)=\frac{\exp\left(\sum_{y\in N\left(x\right)}\delta \left(k-H\left(y\right)\right)\right)}{\sum_{h=1}^{K}\exp\left(\sum_{y\in N\left(x\right)}\delta \left(h-H\left(y\right)\right)\right)}$$ (41)

where *δ*(⋅) stands for the Kronecker's delta function and is given as:

$$\delta \left(k-H\left(y\right)\right)=\{\begin{array}{cc} 1, & \mathrm{if}\;k=H\left(y\right)\\ 0, & \mathrm{otherwise} \end{array}$$ (42)

In this section, we compared our method with other methods on synthetic and clinical brain MR images.

### 3.1 Evaluation with 3T Brain MR images

**Fig 2:**
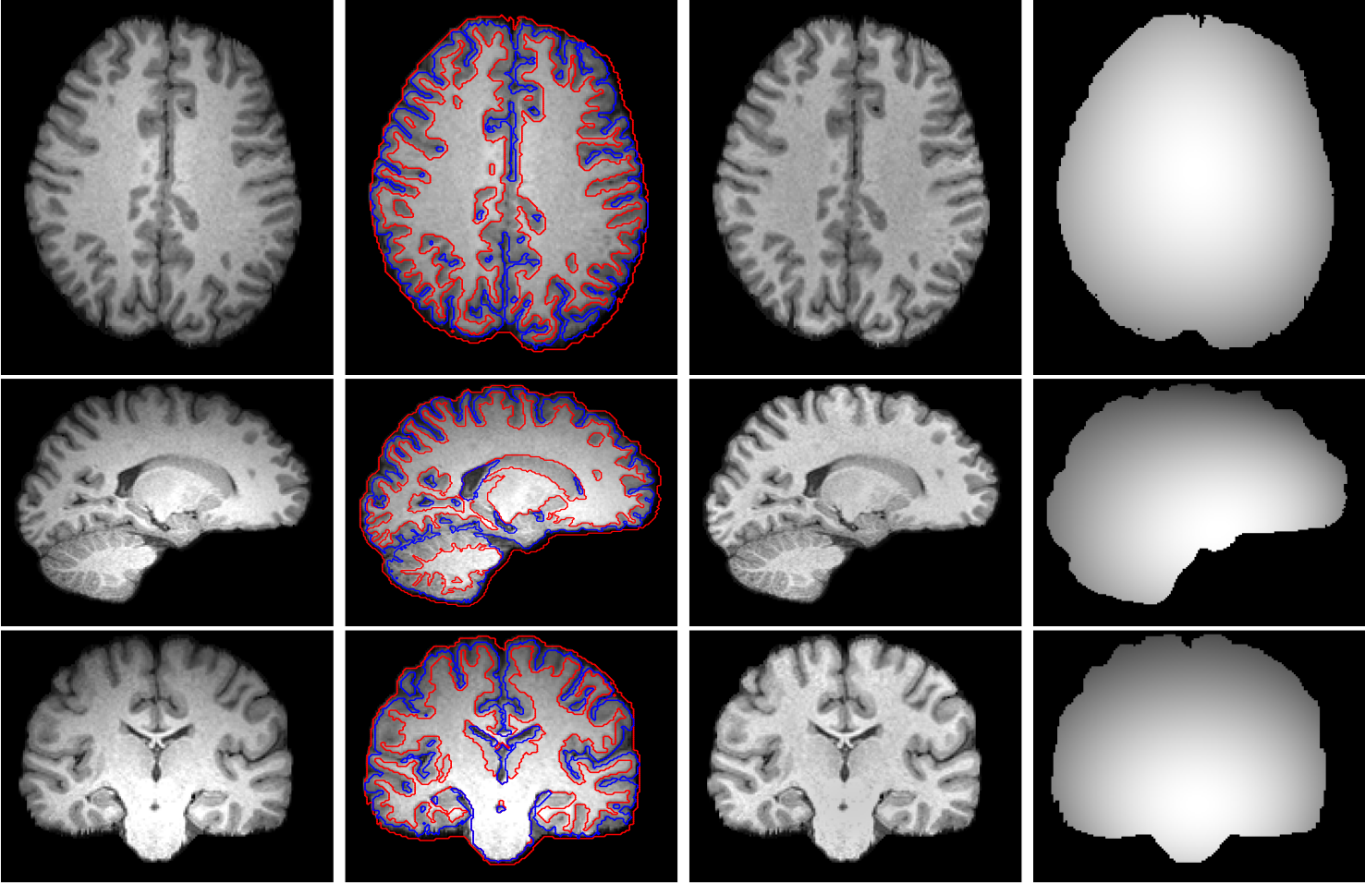
Illustration of (1st column) three 3-Tesla brain MR images, (2nd column) segmentation results of the proposed method, (3rd column) bias corrected images, and (4th column) their estimated bias fields.

We first test our method on three 3-Tesla brain MR images (show n in the 1st column of Fig 2), which is corrupted with severe intensity inhomogeneity. The segmentation results, bias corrected images and estimated bias fields are shown in Fig 2. It can be found that the intensities in each brain tissue of the bias corrected images become quite homogeneous and our method can obtain satisfied results even on weak edges. It demonstrates that the results of our method are consistent with the expected tissue regions.

**Fig 3:**
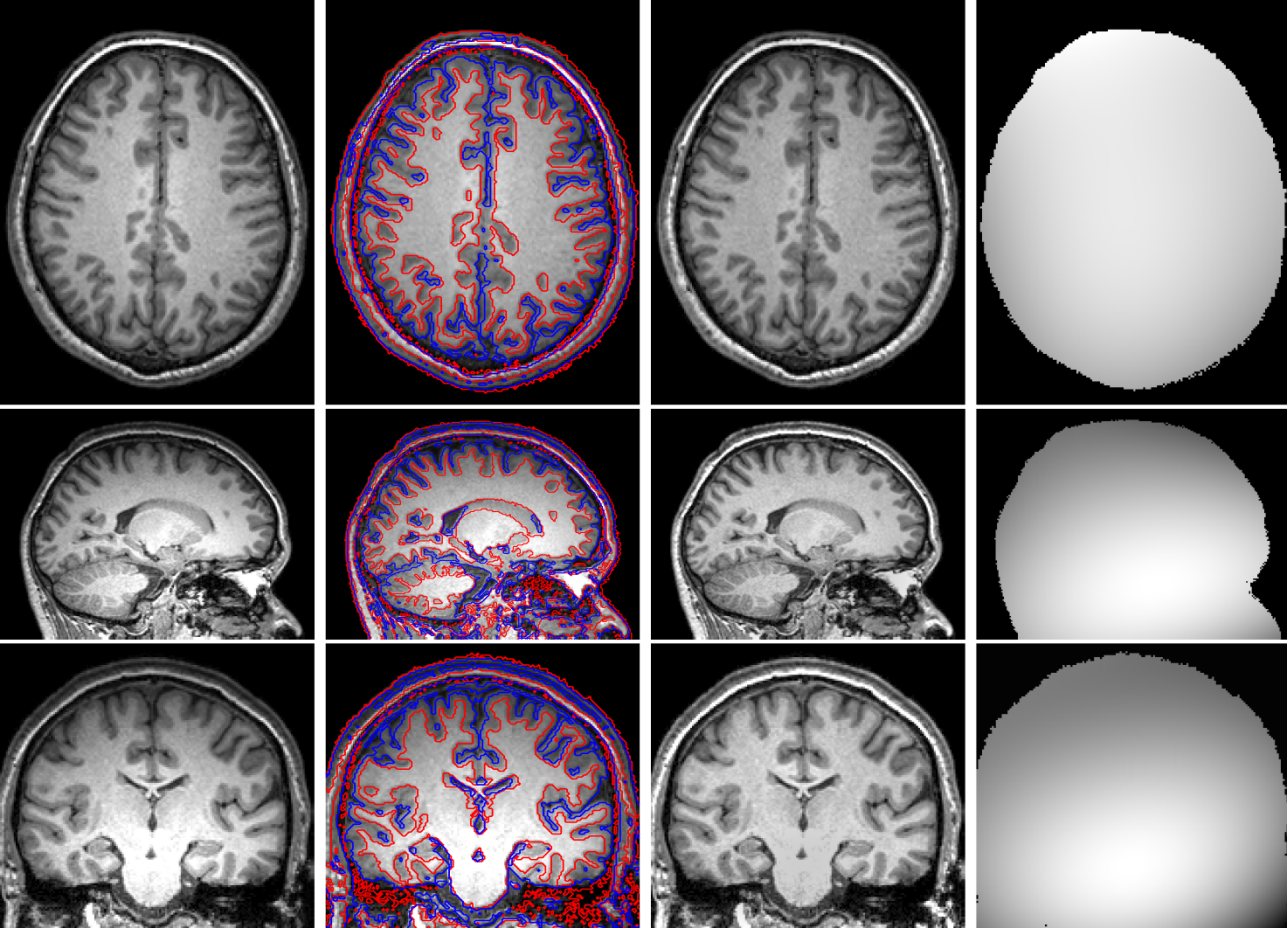
Illustration of (1st column) three 3-Tesla brain MR images with skull, (2nd column) segmentation results of the proposed method, (3rd column) bias corrected images, and (4th column) their estimated bias fields.

Non-brain tissues usually affect the accuracy of the segmentation methods [36], in order to demonstrate the ability of our method, we test our method on three 3-Tesla brain MR images with skulls. The initial images, segmentation results, bias corrected images and the estimated bias fields are shown form left to right in Fig 3. It is clear that our method can still obtain satisfactory results without being influenced by non-brain tissues.

### 3.2 Quantitative comparison

In this section, we quantitatively compared the proposed method to three existing segmentation approaches, including the improved LBF method [37], the improved LGD method [17] and Zhang's method [27]. Generally, the parameters for each method are set with the default values specified in the papers. Please refer to the corresponding references for more details. To make a fair comparison, the curves are initialized by using k-means method.

**Fig 4:**
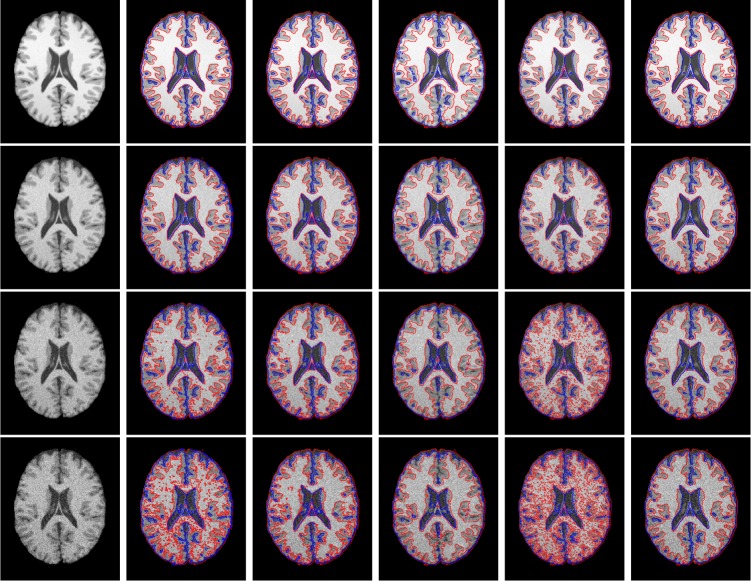
Segmentation results on the 92th transaxial image of a synthetic image data set. The first column shows the initial images with parameters: noise level 1%, 3%, 5% and 7% (from top to the bottom), respectively. The images have the same intensity inhomogeneity level: 30%. The second column to the right column show the segmentation results of improved LBF method, improved LGD method, Zhang's method, MICO and our method, respectively.

All these methods can reduce the impact of noise and intensity inhomogeneity. Therefore, we first apply all the methods on the synthetic brain MR images selected from Brain Web containing different levels of noise and the same intensity inhomogeneity. In our experiments, we use the T1-weighted 1mm brain MR images. Fig 4 shows the segmentation results on the 92th transaxial image of a synthetic image data set from Brain Web. The first column shows the initial images parameters: noise level 1%, 3%, 5% and 7% (from top to the bottom), respectively. The images have the same intensity inhomogeneity level: 30%. The second column to the right column show the segmentation results of the improved LBF method, improved LGD method, MICO (Multiplicative intrinsic component optimization method), the Zhang's method and our method, respectively.

The improved LBF method uses the local intensity mean to fit the intensity of local intensity distribution and thereby achieves much better segmentation results than those of traditional active contour methods, i.e. PS method. However, this method has not considered any spatial information, which makes the method still sensitive to noise when the noise level is high. From the results shown in the second column of Fig 4, we can find that the accuracy of the improved LBF method decreases when the noise level is increasing. In order to reduce the effect of noise, the method can change the control parameter of the length term; however, when the control parameter increases, the length term will makes the method lose detail information. The improved LGD method uses a Gaussian distribution to fit the intensities in each local region and can achieves much better segmentation results than the improved LBF method. Similar drawbacks of the improved LBF method still exist for the improved LGD method, which has not any spatial information been considered. It can be seen from the results shown in the third column of Fig 4 that the improved LGD method is less sensitive to the noise than that of the improved LBF method, but still sensitive to noise. The Zhang's method sets the variances of Gaussian distribution to be a piecewise constant in each region to improve the accuracy of the improved LGD method. Furthermore, the Zhang's method uses a constant kernel to indicate the local region, which is based on a solid theoretical foundation and can reduce the effect of noise. However, the constant kernel easily makes the method lose details. From the results of Zhang's method shown in the fourth column of Fig 4, we can find that the method can reduce the effect of noise but at the same time it loses some details. The MICO method [33] uses the Legendre orthogonal polynomials to model the bias field, however, the method has not considered any spatial information, which makes the method sensitive to noise and the results can be found in the fifth column of Fig 4. From the results, we can found that the accuracy is decreasing when the noise level is increasing.

**Fig 5:**
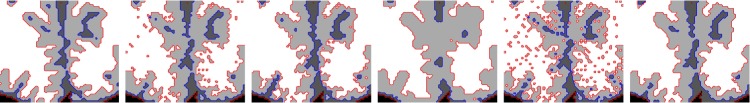
Details of the segmentation results on the 92th transaxial image of a synthetic image data set with parameters: noise level 5% and the intensity inhomogeneity level: 30%. The second column to the right column show the segmentation results of improved LBF method, improved LGD method, Zhang's method, MICO and our method, respectively.

In order to illustrate the problem more clearly, we magnified part of the segmentation results of all the four methods on the image with noise level 5% and intensity inhomogeneity level 30%. The details are shown in Fig 5. From left to right show the details of the ground truth, the result of the improved LBF method, the improved LGD method, the Zhang's method, MICO and our method, respectively. It can be found that the improved LBF method, the improved LGD method and MICO are sensitive to noise, Zhang's method has mis-segmented some pixels belong to CSF into GM. Comparing with ground truth and segmentations obtained with other algorithms, the proposed method can visually obtain the best results.

**Fig 6:**
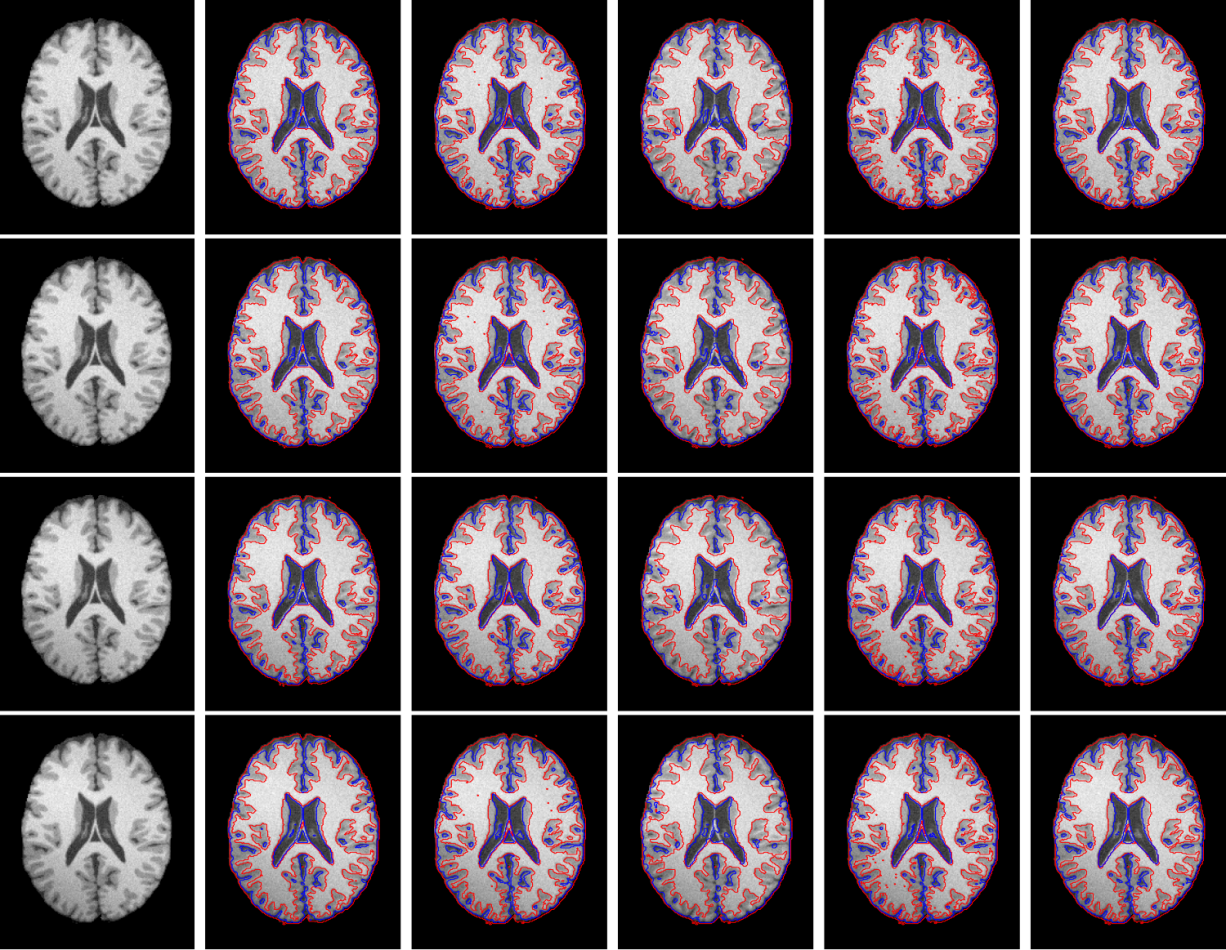
Segmentation results on the 92th transaxial image of a synthetic image data set. The first column shows the initial images with parameters: intensity inhomogeneity level 40%, 60%, 80% and 100% (from top to the bottom), respectively. The images have the same noise level: 3%. The second column to the right column show the segmentation results of improved LBF method, LGD method, Zhang's method, MICO and our method, respectively.

In order to show the robustness on the images with the intensity inhomogeneity, we compared our method with the above three methods on the 92th transaxial image of a synthetic image data set from BrainWeb. The initial images with parameters: intensity inhomogeneity level 40%, 60%, 80% and 100% (from top to the bottom in Fig 6), respectively. The images have the same noise level: 3%. The improved LBF method has considered the bias field when utilizing the local intensity means to fit the intensity of local intensity distribution and can estimate the bias field when segmenting images. The improved LBF method uses a Gaussian kernel as the local spatially weighted function to control the radius of local region, which also preserves the smoothness of the bias field. In order to obtain more accurate result, the radius of the Gaussian kernel cannot be set too large, which makes the estimated bias field inaccurate when the intensity inhomogeneity in the image is severe. Form the results of the improved LBF shown in the second column of Fig 6, we can find that the accuracy decrease s when the intensity inhomogeneity level increases. Similar drawbacks exist for the improved LGD method (see the third column of Fig 6). Compared with the Gaussian kernel used in the improved LBF method and improved LGD method, the Zhang's method uses a constant kernel to indicate the local region. The constant kernel can reduce the effect of noise; however, as analyzed above, the constant kernel also may lose details. The estimation of the bias field in all these three methods are based on local region information, which makes these methods easily be affected by the intensities of the pixels in each local region and cannot be smoothed enough. MICO uses the Legendre orthogonal polynomials to model the bias field, which makes it possible for the method to obtain more smoothly bias fields. From the result, we can still find that the method is sensitive to noise.

**Fig 7:**
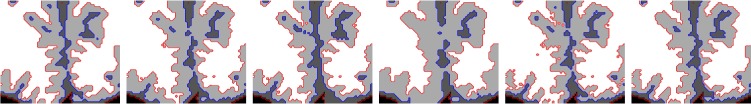
Details of the segmentation results on the 92th transaxial image of a synthetic image data set with parameters: noise level 3% and the intensity inhomogeneity level: 80%. The second column to the right column show the segmentation results of improved LBF method, LGD method, Zhang's method, MICO and our method, respectively.

From the results shown in Fig 6, it can be found that the accuracies of all these three are affected when the intensity inhomogeneity level increases. In order to illustrate the problem more clearly, we magnified part of the segmentation results o f all the four methods on the image with noise level 3% and intensity inhomogeneity level 80% and the details are shown in Fig 7. From left to right show the details of the ground truth, the result of improved LBF method, improved LGD method, Zhang's method, MICO and our method, respectively. It can be found that comparing with the ground truth and segmentations obtained with other algorithms, the proposed method can visually obtain the best results.

For quantitative comparison, we use Js values as a metric to evaluate the performance of these methods. The Js is defined as the ratio between intersection and union of two sets *S*_1 and *S*_2

$$J_{S}\left(S_{-}1,S_{-}2\right)=\frac{\left|S_{-}1\cap S_{-}2\right|}{\left|S_{-}1\cup S_{-}2\right|}$$ (43)

where *S*_1 is the segmentation result and *S*_2 is the ground truth. A more accurate result should have a higher Js value. In order to show the robustness and accuracy of our proposed method, we applied the above three methods and our method on whole simulated MRI data sets from BrainWeb with different noise levels and intensity inhomogeneity levels. The accuracy is measured by using the means and standard deviations of Js values on WM, GM and CSF. The average results are listed in Table 1. In this table, we set *N*_*x*_*F*_*y*_ as image data with noise level *x*% and intensity inhomogeneity level *y*%. From the values, it can be found that our proposed method has the best means of Js values, which indicates that our method is more accurate than other methods. It also can be found that the standard deviations of our method are much lower than the others, which proved that our method has the best robustness. We also find that our method has the highest mean Js values of CSF, which illustrates that our method can preserve more details.

**Table 1:** Js values (mean ± standard deviation) for the segmentation results on simulated T1-weighted brain MR images. (%).

Image	Tissues	ILBF [37]	ILGD [17]	Zhang's [27]	MICO [33]	Our method
N1F30	WM	93.62±4.78	95.21±3.75	92.83±3.05	93.77±3.09	96.33±2.27
	GM	92.09±3.92	92.14±3.06	91.35±4.04	92.41±2.91	93.52±3.06
	CSF	91.97±3.84	92.10±2.51	90.21±4.24	92.14±3.25	93.21±1.92
N3F30	WM	92.46±4.25	93.95±3.95	91.96±4.84	92.01±4.33	95.76±2.31
	GM	91.52±3.58	92.10±3.53	91.14±3.20	91.10±3.77	93.27±2.07
	CSF	91.09±3.92	91.89±3.42	89.23±4.16	89.77±4.75	93.09±2.30
N3F60	WM	92.39±4.73	93.89±3.07	91.93±4.36	91.67±3.41	95.70±2.72
	GM	91.43±4.09	92.00±3.93	91.06±3.68	91.01±4.22	93.26±2.63
	CSF	91.01±3.21	91.79±4.03	89.16±3.77	89.43±3.66	93.10±2.67
N3F100	WM	92.21±4.92	93.81±3.33	91.89±3.96	91.48±3.01	95.68±3.01
	GM	91.30±5.19	91.14±3.17	91.02±4.03	90.40±2.72	93.21±2.42
	CSF	89.63±4.06	91.46±4.95	89.11±4.88	89.25±4.01	92.99±2.93
N5F30	WM	89.43±6.24	91.09±4.96	91.27±5.03	87.25±3.69	94.07±4.03
	GM	87.26±3.57	89.33±4.61	90.52±4.06	86.38±5.21	92.28±2.80
	CSF	86.75±3.07	88.69±6.27	89.27±5.14	85.01±6.39	92.03±1.92
N7F30	WM	78.68±10.02	87.82±8.66	90.06±4.58	75.58±9.95	93.27±5.12
	GM	72.49±5.71	86.60±4.23	88.27±5.07	70.22±11.25	92.43±2.91
	CSF	78.34±6.07	87.09±3.97	87.96±3.63	72.66±8.77	91.12±3.30

**Fig 8:**
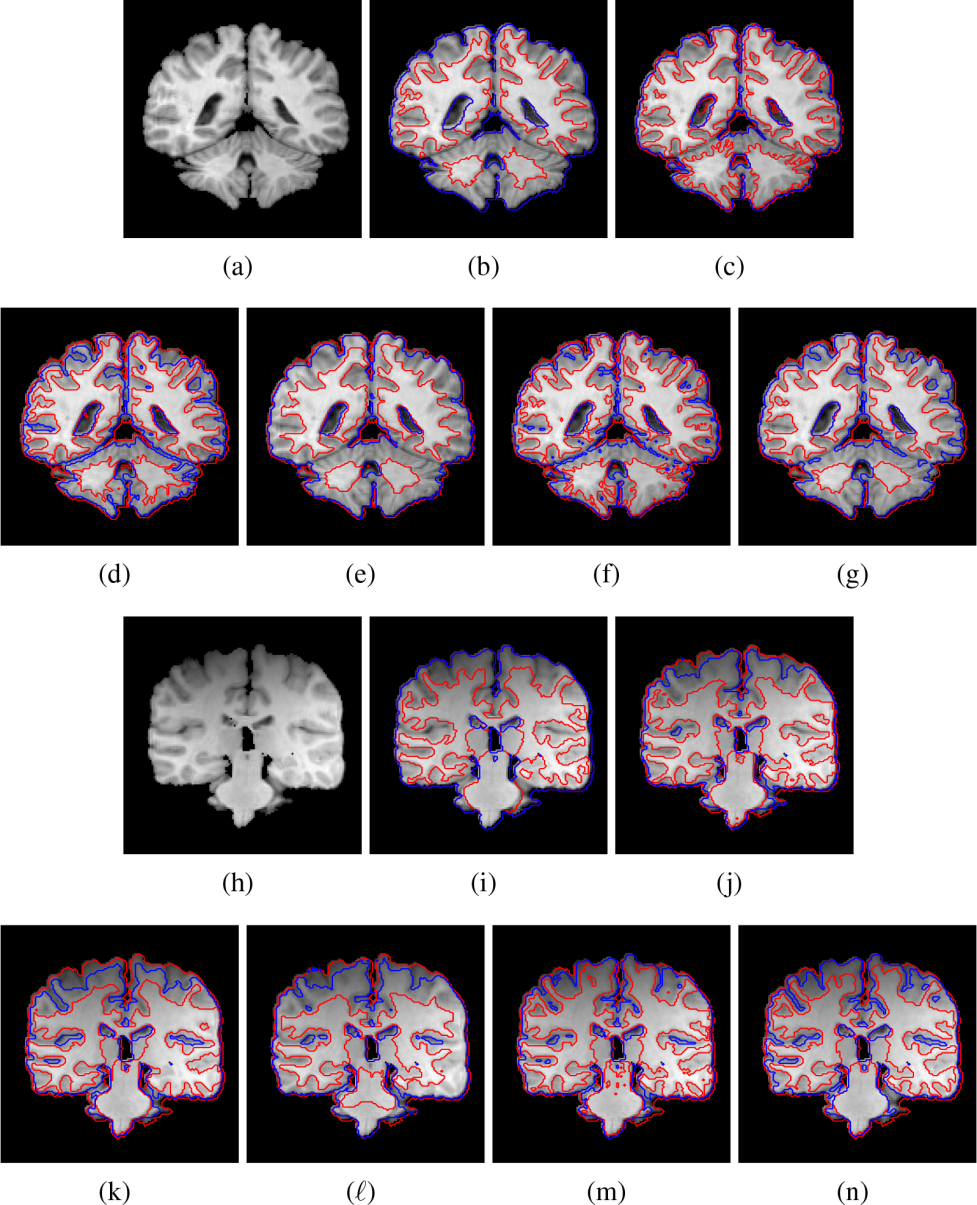
Segmentation results on clinical brain MR images. (a) is the 18th image of 1_24♯, (b)-(g) show the ground truth, the segmentation results of improved LBF method, improved LGD method, Zhang's method, MICO and our method, respectively. (g) is the 30th image of 2_4♯. (i)-(n) are the ground truth, the segmentation results of improved LBF method, improved LGD method, Zhang's method, MICO and our method, respectively.

In the next experiment, we compare the proposed method to above three segmentation methods on clinical T1-weighted brain MR images selected from IBSR. Fig 8(A) shows the 18th image from IBSR (1_24♯) and the corresponding ground truth is shown in Fig 8(B). It can be found that Fig 8(A) has low contrast and severe intensity inhomogeneity. Due to the effect of the severe intensity inhomogeneity, the improved LBF method (ILBF) trapped into local optima and mis-segmented some pixels belong to GM into WM, which can be found in Fig 8(C). The improved LGD method can also obtain more accurate result by using local Gaussian distribution to fit the local intensity distribution. However, the improved LGD method used local variance information, which is unstable [27] and makes the method mis-segmented some pixels belong to GM into WM. The Zhang's method used the piecewise constant variances of the Gaussian distributions in each region and can preserve some details. However, the method uses constant kernel to convolution, which makes the method hard to find accurate results when segmenting images with low contrast. Form the result shown in Fig 8(E), we can find that Zhang's method has lost some details. Fig 8(F) shows the segmentation result of MICO. The MICO uses global means information of the intensities, which makes the method sensitive to low contrast and mis-segmented some pixels belong to GM into WM. Fig 8(H) shows the 30th image from IBSR (2_4♯), which has intensity inhomogeneity and severe low contrast. The ground truth is shown in Fig 8(I). The improved LBF method only uses the local mean information, which makes the method sensitive to low contrast. Similar drawbacks can be found in the improved LGD, zhang's method and MICO. In our method, we use anisotropic patch information and multivariate Student's t-distribution to fit the intensity distribution to improve the accuracy. Furthermore, the bias field is estimated by using basis functions, which makes our method more robust to severe intensity contrast. The average mean of JS values of the eight methods on the whole data sets (1_24♯ and 2_4♯) form IBSR are listed in Table 2. Because there are only small pixels be-long to CSF are contained in IBSR data sets, we only list the Js values for WM and GM. From the average Js values shown in Table 2, we can find that our method obtains the most accurate results.

**Table 2:** Js values (mean ± standard deviation) for the segmentation results on simulated T1-weighted brain MR images. (%).

Imgae	Tissues	ILBF [37]	ILGD [17]	Zhang's [27]	MICO[33]	Our method
1_24♯	WM	76.98±8.26	84.04±8.00	82.06±5.43	78.01±9.11	88.04±2.92
	GM	62.67±10.39	71.54±5.64	76.82±8.01	61.88±9.01	78.29±3.01
2_4♯	WM	73.86±7.64	74.28±5.28	62.18±5.07	75.85±6.98	82.87±4.55
	GM	61.75±8.88	60.46±7.02	53.85±3.32	63.52±8.25	72.52±3.67

**Fig 9:**
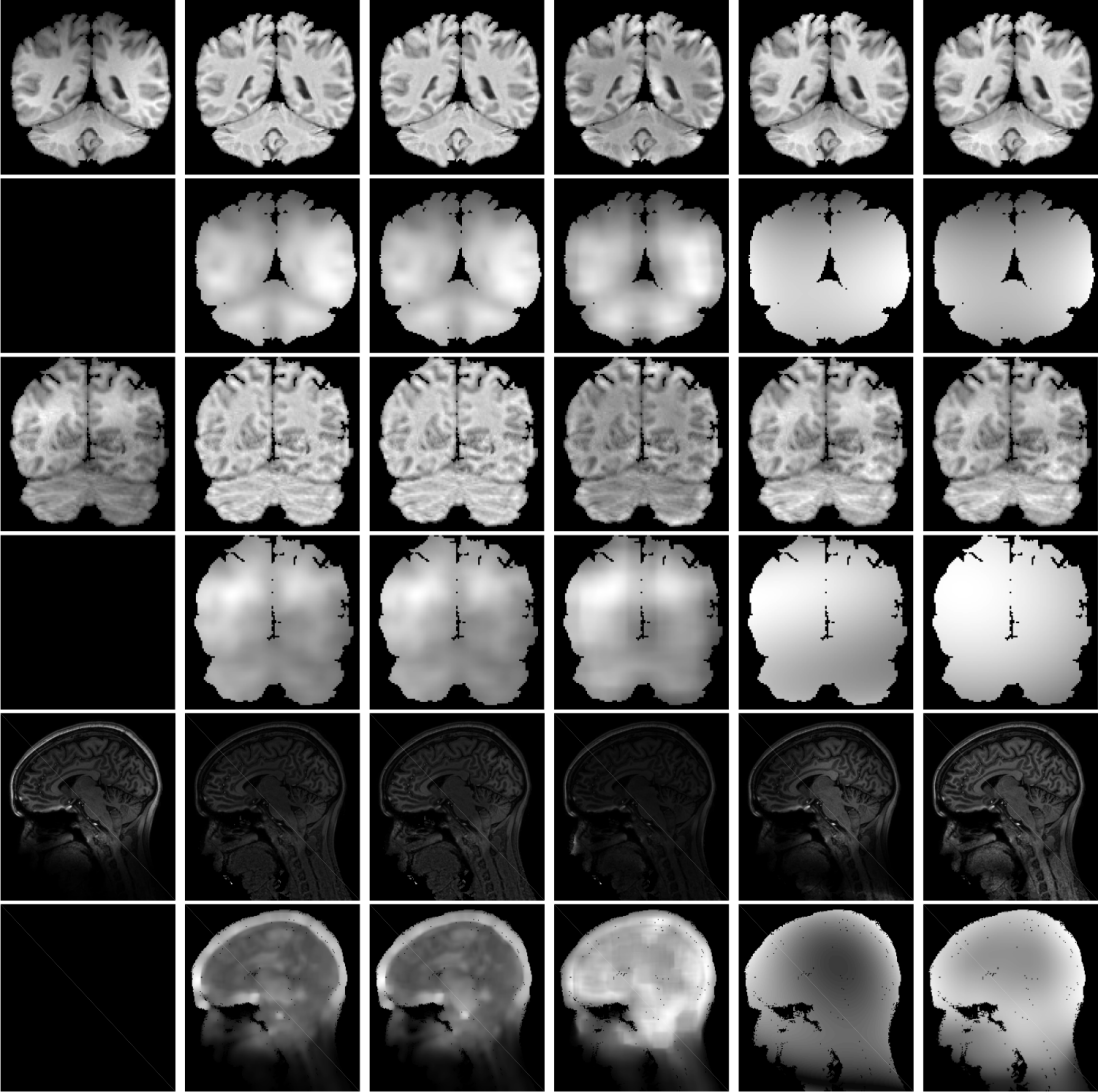
Illustration of two 3-T intensity inhomogeneity corrupted brain MR images and one 7-T brain MR images (1st column). The second column to right column show the results of improved LBF, improved LGD, Zhang's method, MICO and our method, respectively. The odd rows show the bias field corrected images. The even rows show the corresponding estimated bias fields.

In the next experiment, we use coefficient of variance (CV) as a measure to evaluate the performance of the algorithms for intensity inhomogeneity correction [35]. The CV is defined as a percentage and calculated from the average and standard deviation of selected tissue. A good algorithm can obtain low CV values for the bias corrected intensities within each segmented region. We compared our method with improved LBF method, improved LGD method and Zhang's method on two 3-T brain MR images with intensity inhomogeneity (first one is from IBSR 2_4♯ and another one is from 15_3♯) and a 7-T brain MR image, which has severe intensity inhomogeneity. Fig 9 shows the bias field corrected images and the corresponding bias field. The second column to right column shows the results of improved LBF method, improved LGD method, Zhang's method, MICO and our method, respectively. Form the results, it can be found that the bias fields estimated by using the improved LBF method, improved LGD method and Zhang's method are not smooth enough. That is because all three method use convolution to preserve the smoothness of the methods. MICO and our method can obtain more smooth and accurate bias filed by using the basis functions to model t he bias field. To make a fair comparison, the segmentation results are obtained by using Fuzzy C-means Clustering method when calculating the CV values. The values of CV are listed in Table 3. It can be seen that our method can obtain the smallest CV values, which indicates that the bias corrected images obtained by using our method are more homogeneous than others.

**Table 3:** CV values for the bias corrected images. (%).

Imgae	Tissues	ILBF [37]	ILGD [17]	Zhang's [27].	MICO[33]	Our method
2_4♯	WM	7.26	6.87	10.74	6.35	6.17
	GM	17.72	17.19	16.08	16.27	16.02
15_3♯	WM	7.72	7.70	10.11	6.82	6.74
	GM	13.31	13.32	12.42	12.34	12.17
7-T	WM	23.54	19.52	19.25	19.21	19.14
	GM	29.19	24.35	23.32	22.59	20.92

Our model is also superior in terms of computational efficiency. The mean CPU times for 20 simulated MR image data sets and 20 standard sets of real brain MR data are listed in Table 4, which were recorded from our experiments with Matlab code run on a Lenovo computer, with i7 processor, 2.40 GHz, 7.88G RAM, with Matlab 7.1 on Windows10. The sizes of these images are also shown in this table. The computation time of our method was less than 1 sec on 2D images and less than 4 minutes on 3D images, which is much faster than the other methods. This demonstrates the significant advantage of our model in terms of computational efficiency.

**Table 4:** CPU time (in second) for the segmentation.

Imgae	ILBF [37]	ILGD [17]	Zhang's [27].	MICO[33]	Our method
181 × 181	112.24	0.68	3.52	0.32	0.70
181 × 217	115.82	0.73	3.84	0.43	0.76
256 × 256	132.57	0.86	4.27	0.47	0.87
256 × 256 × 171	4280.25	275.68	-	428.81	292.64

### 3.3 Segmentation on 3D image data

**Fig 10:**
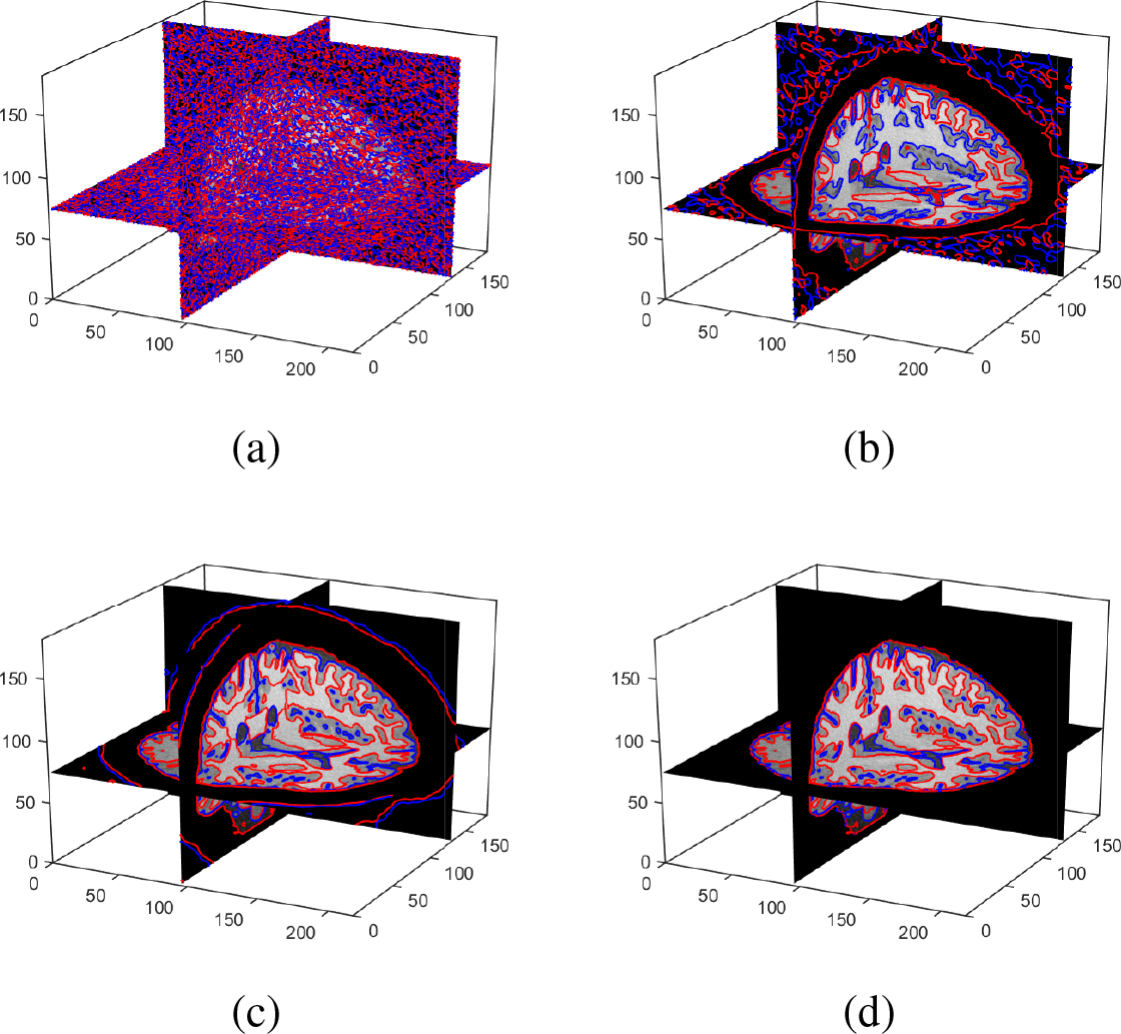
3D segmentation results of WM, GM and CSF on T1-weighted 1mm brain MR images from BrainWeb with parameters: noise level 3% and intensity inhomogeneity level: 80%. (b)-(d) show the results of the 3rd iteration, 5th iteration and 7th iteration, respectively.

**Fig 11:**
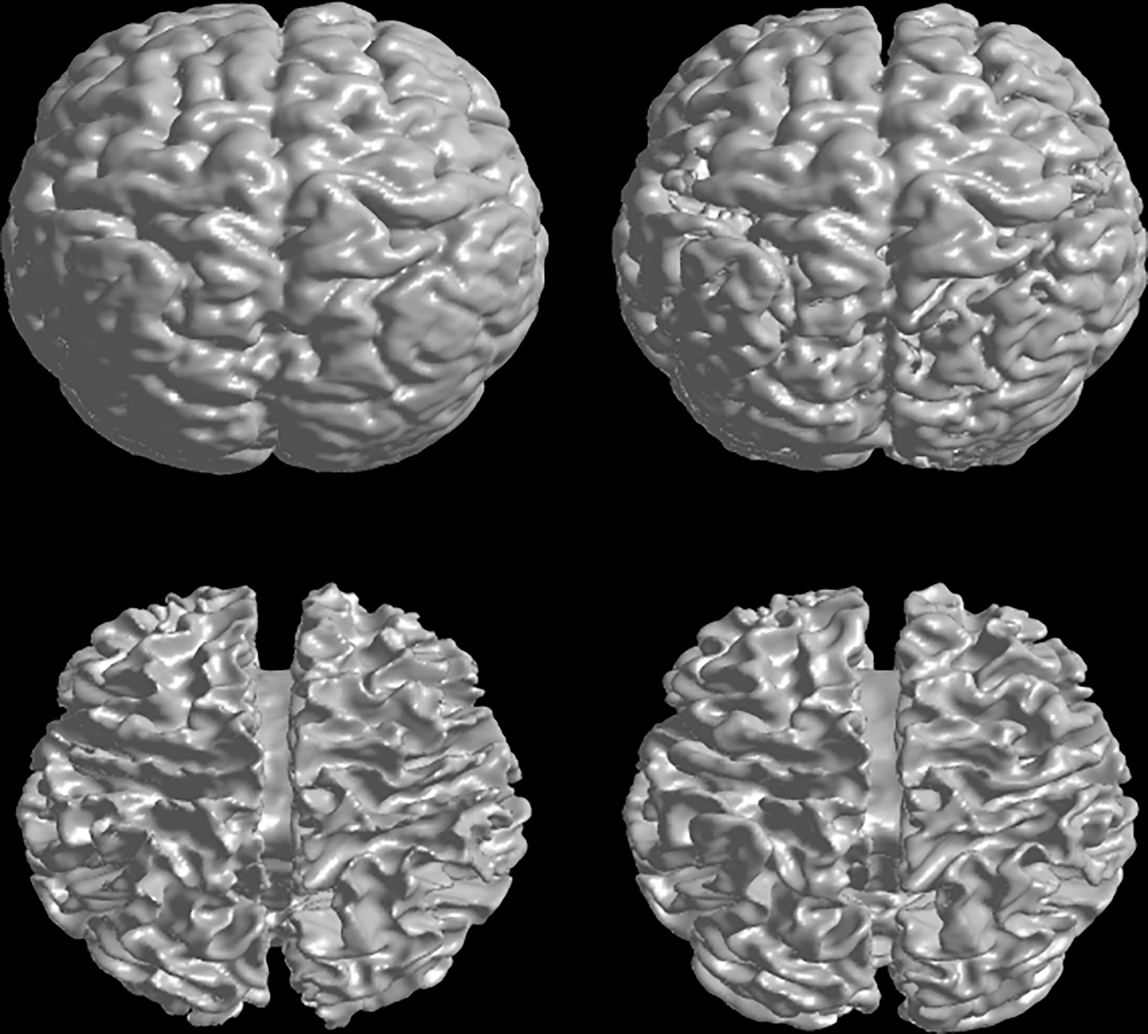
3D segmentation results of WM, GM on T1-weighted clinical brain MR image. Left column shows the ground truth and the right column shows the results of our method.

In this section, we test our method on 3D image data. Fig 10 shows the segmentation results of our method for the BrainWeb Data with noise level 3%, intensity inhomogeneity level 80%. In this experiment, the initial surfaces are initialized randomly, which can be seen in Fig 10(A). In order to illustrate the evolutions of the surfaces, we presented the corresponding contour evolution of three slices in different axis for the 3rd, 5th and 7th iteration results. From the results, we can find that our method can obtain the satisfactory result in 7 iterators even with randomly initialization. Fig 11 shows the segmentation of our method on a 3D clinical brain MR image from ISBR 2.0(7#), which has intensity inhomogeneity and low contrast. The left column shows the ground truth of GM and WM. The right column shows the segmentation results of our method. From the results, we can find that our method can obtain the satisfactory result.

## Discussion and conclusion

In this paper, we proposed a novel automatic variational level set based segmentation and bias field estimation method for human brain MR images. This method successfully overcomes the drawbacks of existing active contour methods, including limited robustness to weak edges, noise, intensity inhomogeneity and limited accuracy to details, by proposing improved anisotropic spatially information to reduce the effect of noise and preserve more details; utilizing multivariate Student's t-distribution to fit the intensity distribution of the image to improve the robustness; using the HMRF to construct data term to incorporate more spatial information. In order to obtain more accurate and smoothed bias field, we use a linear combination of orthogonal basis functions to model the bias field. To find the global optimal and make the results independent of the initialization of the algorithm, we reconstructed the energy function to be convex and calculated it by using the Split Bregman algorithm. Our statistical results on both synthetic and clinical images show that the proposed method can overcome the difficulties caused by noise, weak edges and intensity inhomogeneity, and outperforms other several state-of-the-art methods.

The degree of basis function determines the accuracy and stability of the bias field correction. A lower degree will make the estimated bias field inaccurate when the intensity inhomogeneity is severe. A large degree will make our method inefficient, unstable and easily trap into local optima. Our experiments showed that the degree of basis functions up to 4 sufficiently models the bias field.

One challenge of the method presented in this paper is how to set the parameter *β* in Eq (15). The *β* depends on the size of neighbor patch. A much bigger *β* will make the weights of the other pixels in neighbor patch be smaller than that of the center pixel, which will decrease the robustness to noise. On the other side, a much smaller *β* will make the weights of all pixels in the neighbor patch be similar, which makes the neighbor information isotropic and lose details. In most regions, the intensity distances between the neighbor pixels to the center are always less than a certain threshold. After experimented on more than 100 data sets, we found that the results are accurate enough for the *β* = 0.02 when the patch size is 3×3. One possible extension of this work is to optimize *β* throughout the image automatically. Moreover, the selection of the patch size should be set based on the amount of noise and the details in the image. Improving the method with adaptive patch size selection will be another direction in the future of work.

Since the magnetic resonance imaging technology has been proposed, it has been widely used to analyze the pathologies. For brain pathologies, tumors, Alzheimer disease, Parkinson, etc., can be treated, the important open questions for brain MR image segmentation methods are how to find the boundaries of the special tissues, such as the hippocampal, amygdala and basal ganglia, which have similar intensities with their neighbor tissues. In order to deal with this problem, many approaches have been suggested to utilize multi-modality information [54], registration methods [55], etc. However, the use of multi-modality or atlas information makes the methods more complex. Thus, addressing such questions is out of the scope of this paper and subjects of future research.

## Supporting information

S1 File The initial images of Fig 2. (ZIP)

S2 File The initial images of Fig 3. (ZIP)

S3 File The initial images of Fig 4. (ZIP)

S4 File The initial images of Fig 6. (ZIP)

S5 File The initial images of Fig 8. (ZIP)

S6 File The initial images of Fig 9. (ZIP)

S7 File The initial images of Fig 10. (ZIP)

S8 File The initial images of Fig 11. (ZIP)
