# Supplementary figures and images for: Brain MR image segmentation based on an improved active contour model
Source: PLoS One. 2017 Aug 30;12(8):e0183943. doi: 10.1371/journal.pone.0183943 (PMC5576762; doi:10.1371/journal.pone.0183943)

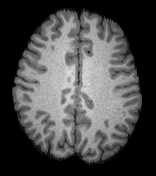

Supplement: S1 File — (ZIP) [file pone.0183943.s001.zip › fig.2/first line.bmp]

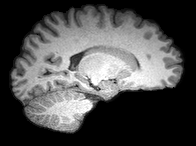

Supplement: S1 File — (ZIP) [file pone.0183943.s001.zip › fig.2/second line.bmp]

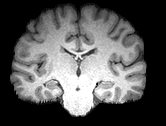

Supplement: S1 File — (ZIP) [file pone.0183943.s001.zip › fig.2/third line.bmp]

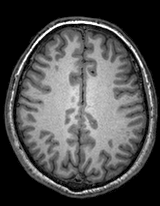

Supplement: S2 File — (ZIP) [file pone.0183943.s002.zip › fig.3/first line.bmp]

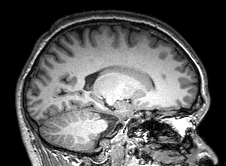

Supplement: S2 File — (ZIP) [file pone.0183943.s002.zip › fig.3/second line.bmp]

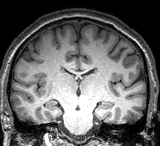

Supplement: S2 File — (ZIP) [file pone.0183943.s002.zip › fig.3/third line.bmp]

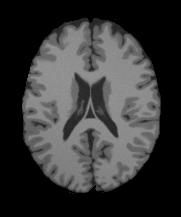

Supplement: S3 File — (ZIP) [file pone.0183943.s003.zip › fig.4/192_N1F30.bmp]

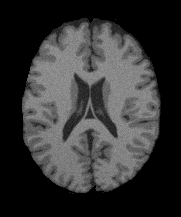

Supplement: S3 File — (ZIP) [file pone.0183943.s003.zip › fig.4/192_N3F30.bmp]

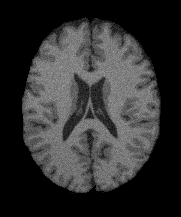

Supplement: S3 File — (ZIP) [file pone.0183943.s003.zip › fig.4/192_N5F30.bmp]

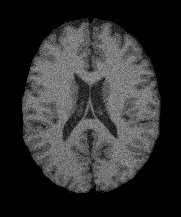

Supplement: S3 File — (ZIP) [file pone.0183943.s003.zip › fig.4/192_N7F30.bmp]

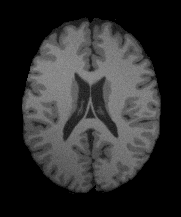

Supplement: S4 File — (ZIP) [file pone.0183943.s004.zip › fig.6/192_N3F100.bmp]

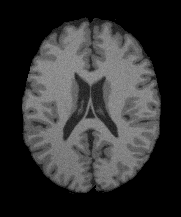

Supplement: S4 File — (ZIP) [file pone.0183943.s004.zip › fig.6/192_N3F40.bmp]

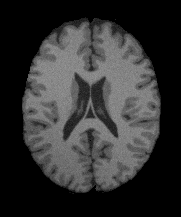

Supplement: S4 File — (ZIP) [file pone.0183943.s004.zip › fig.6/192_N3F60.bmp]

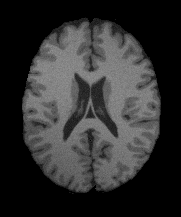

Supplement: S4 File — (ZIP) [file pone.0183943.s004.zip › fig.6/192_N3F80.bmp]

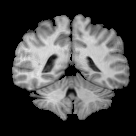

Supplement: S5 File — (ZIP) [file pone.0183943.s005.zip › fig.8/118.bmp]

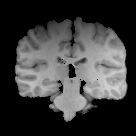

Supplement: S5 File — (ZIP) [file pone.0183943.s005.zip › fig.8/130.bmp]

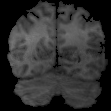

Supplement: S6 File — (ZIP) [file pone.0183943.s006.zip › fig.9/113_15_3.bmp]

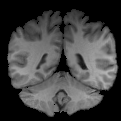

Supplement: S6 File — (ZIP) [file pone.0183943.s006.zip › fig.9/125_2_4.bmp]

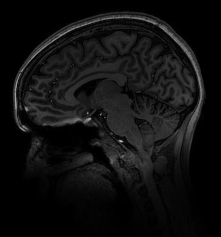

Supplement: S6 File — (ZIP) [file pone.0183943.s006.zip › fig.9/image7T_EAS_sag92.bmp]
